# Piezo1-mediated mechanotransduction and metabolic regulation in bone health: molecular mechanisms and implications for bone disorders

**DOI:** 10.3389/fcell.2026.1839596

**Published:** 2026-06-24

**Authors:** Jianbo Feng, Changyong Ye, Yun He, Fuyin Yang, Jinglin Li

**Affiliations:** 1 Department of Orthopedics, The People’s Hospital of Zheng’an, Zunyi, China; 2 Department of Orthopedics, Affiliated Hospital of Zunyi Medical University, Zunyi, China; 3 Department of Anatomy, Zunyi Medical and Pharmaceutical College, Zunyi, China; 4 Department of Orthopedics Center, Renhuai People’s Hospital, Zunyi, China; 5 Joint Orthopaedic Research Center of Zunyi Medical University, University of Rochester Medical Center, Zunyi, China

**Keywords:** bone homeostasis, cell fate, fracture healing, intervertebral disc degeneration, mechanotransduction, osteoarthritis, osteoporosis, Piezo1

## Abstract

Bone continuously adapts to mechanical forces to maintain structural integrity, yet the molecular sensors that initiate this process have long remained undefined. The identification of the mechanosensitive ion channel Piezo1 has provided a pivotal molecular basis for understanding skeletal mechanotransduction. This review summarizes current advances in elucidating the unique structural features and force-gating mechanisms of Piezo1, and highlights its role as a central mechanoreceptor coordinating mechanical responses within bone tissue. We further delineate the multidimensional downstream signaling networks activated by Piezo1 and discuss the complex crosstalk among these pathways. The pathological consequences of Piezo1 dysregulation in major orthopedic disorders are examined, along with the therapeutic potential and challenges of targeting Piezo1 as a novel “mechanopharmacological” strategy. Collectively, this review provides an integrated framework for understanding the molecular foundations of bone mechanotransduction and identifies Piezo1 as a promising target for developing innovative treatments for orthopedic diseases.

## 1 Introduction

As the primary load-bearing organ of the human body, the skeleton possesses remarkable structural integrity and adaptive capabilities that are fundamentally shaped and maintained by its mechanical environment (Morgan et al., 2018). As early as the 19th century, German anatomist Julius Wolff articulated the seminal Wolff’s Law, which precisely encapsulates the dynamic relationship between bone morphology and functional loading: bone undergoes adaptive remodeling in response to changes in the stress patterns it experiences (Kao et al., 2019; Chen X. et al., 2025). This principle not only explains the maintenance of skeletal homeostasis but also underscores the decisive role of mechanical loading in bone development, growth, and repair (Sui et al., 2024). While moderate mechanical stimulation promotes osteogenesis and increases bone mineral density, a chronic lack of mechanical loading—such as in microgravity environments (spaceflight) or prolonged bed rest—leads to catastrophic bone loss and disuse osteoporosis (Vico and Hargens, 2018; Spatz et al., 2012; Bezerra et al., 2022). Conversely, excessive or aberrant mechanical stress, such as chronic articular overloading, accelerates cartilage degeneration, eventually precipitating degenerative pathologies like osteoarthritis (OA) (Wang and He, 2018). Underlying these macroscopic phenomena is the complex process of mechanotransduction—the conversion of physical force signals into biochemical signals at the cellular level—which serves as the molecular bridge connecting the macroscopic mechanical environment to microscopic cellular behavior (Saraswathibhatla et al., 2023; Wang et al., 2023). Consequently, a profound understanding of the molecular mechanisms governing mechanotransduction in bone tissue is critical for elucidating skeletal physiology, understanding pathogenesis, and developing innovative therapeutic strategies.

Although the concept of mechanotransduction is well-established in bone biology, the precise molecular mechanisms by which cells perceive and respond to mechanical forces have long remained a challenging frontier. Historically, integrins and their associated cytoskeletal components, focal adhesion kinase (FAK), and receptor tyrosine kinases (RTKs) were regarded as the primary mechanosensors (Chen et al., 2023a; Cooper and Giancotti, 2019). These molecules transmit signals such as matrix stiffness and traction forces from the extracellular matrix (ECM) to the intracellular cytoskeleton, thereby initiating downstream signaling pathways (Zhu et al., 2023). However, these classical sensors primarily mediate relatively slow and sustained cellular responses. The specific molecular entities responsible for the skeleton’s rapid, transient responses to mechanical force—particularly those mediated by ionic electrochemical signals—remained elusive (Kefauver et al., 2020). A paradigm shift occurred in 2010 when the Patapoutian lab identified a novel family of mechanically sensitive cation channels through functional screening, naming them Piezo (Savadipour et al., 2023a; Coste et al., 2012; Coste et al., 2010). This groundbreaking discovery filled a critical gap regarding the core mechanosensitive ion channels in mammalian cells. The Piezo family, comprising Piezo1 and Piezo2, can directly respond to diverse physical stimuli—including membrane stretch, fluid shear stress, osmotic pressure variations, and matrix stiffness—and efficiently translate them into electrochemical signals characterized primarily by calcium (Ca^2+^) influx, thereby triggering a cascade of downstream biological effects (Saotome et al., 2018). Due to its unique molecular architecture and extensive expression in bone tissue, Piezo1 has rapidly emerged as a focal point in skeletal mechanobiology.

The discovery of Piezo1 has stimulated considerable research interest in orthopedics, as it closely matches the characteristics of bone as a mechanosensitive organ. Although Piezo1 is not the only mechanosensitive ion channel in skeletal tissues, it deserves dedicated discussion in the context of bone mechanobiology. Piezo2 and TRPV4 also participate in musculoskeletal mechanotransduction, mainly in sensory neurons and chondrocytes, respectively. However, Piezo1 differs from these related mechanosensors in that it is broadly expressed in non-excitable skeletal cells, including bone marrow mesenchymal stem cells, osteoblast-lineage cells, osteocytes, and chondrocytes, and is directly involved in load-induced bone formation and skeletal homeostasis. Genetic evidence further indicates that Piezo1 plays a dominant role in osteoblast-lineage mechanotransduction, whereas Piezo2 may act as a context-dependent partner, particularly in cartilage exposed to high-strain mechanical stimulation (Qin et al., 2021; Steinecker-Frohnwieser et al., 2023). TRPV4 is closely associated with chondrocyte osmo-mechanotransduction and matrix homeostasis; however, Piezo1 has attracted particular attention because of its rapid force-gated calcium influx, unique trimeric membrane-dome architecture, and capacity to coordinate calcium signaling, cytoskeletal remodeling, metabolic adaptation, inflammatory-stress responses, and transcriptional regulation. Recent studies have confirmed the widespread expression and critical function of Piezo1 across various bone-resident cells. In bone marrow mesenchymal stem Cells (BMSCs), Piezo1 activation robustly drives osteogenic differentiation while suppressing adipogenesis, thus playing a pivotal role in lineage commitment during skeletal repair and regeneration (Hu et al., 2023; He et al., 2024; Wang B. et al., 2025). In osteoblasts, Piezo1 serves as a key sensor for mechanical loading, promoting bone formation and mineralization; its osteoblast-specific deletion results in reduced bone mass and impaired osteogenesis (Li et al., 2019; Brylka et al., 2024). As the primary mechanosensory cells of the skeleton, Osteocytes exhibit significantly elevated Piezo1 expression (Li et al., 2021). Fluid shear stress activates Piezo1 in osteocytes to regulate the expression of factors such as Sclerostin, thereby maintaining mechanical homeostasis—a crucial mechanism in preventing disuse osteoporosis (Meslier et al., 2025). Unexpectedly, Piezo1 activation in Osteoclast precursors inhibits differentiation and bone resorption, revealing a novel mechanism by which mechanical force suppresses bone destruction (Shindo et al., 2025). Furthermore, while Piezo1 helps maintain Chondrocyte homeostasis under physiological loading, its hyperactivation under pathological loading (e.g., in OA) triggers apoptosis and matrix degradation, driving disease progression (Brylka et al., 2024; Yu et al., 2025a). Collectively, these findings illustrate Piezo1’s indispensable status as the “central hub” connecting macroscopic skeletal function with microscopic cellular behavior across development, homeostasis, and pathology.

Despite this progress, existing research tends to focus on singular downstream pathways following Piezo1 activation, lacking a systemic elucidation of the dynamic crosstalk and integrative regulation among multiple pathways. This limitation hinders a comprehensive understanding of how Piezo1 acts as a “master switch” to coordinate complex cellular responses. Distinct mechanical input modes and their parameters result in significant differences in Piezo1 activation patterns and the initiation of downstream signaling cascades (Gudipaty et al., 2017; Lin et al., 2019; Li M. et al., 2022). The cellular response to mechanical force is a highly dynamic and multidimensionally integrated process. Piezo1 may act as an integration center that coordinates the activation of multiple signaling axes and thereby generates context-dependent biological outcomes. However, current studies have not yet fully clarified how different mechanical features reshape Piezo1-mediated signaling networks or how these changes ultimately determine cell fate diversity. In this review, we use the term “Piezo1 mechanical signal rheostat” as a qualitative organizing principle rather than a fully established quantitative predictive model. Piezo1 does not simply function as an on–off switch; instead, its activation pattern is influenced by mechanical variables such as stimulus mode, magnitude, frequency, duration, strain rate, matrix stiffness, and spatial confinement. These inputs can be experimentally controlled using fluid shear stress systems, cyclic stretch or compression platforms, tunable hydrogels, atomic force microscopy, ultrasound, and vibration-based stimulation. Corresponding outputs include channel open probability, current amplitude, calcium transient dynamics, cytoskeletal tension, YAP/TAZ nuclear localization, mitochondrial calcium uptake, ATP production, ROS accumulation, NF-κB/NLRP3 activation, and lineage-specific transcriptional programs. Although available evidence supports a graded and context-dependent relationship between mechanical stimulation and Piezo1-dependent signaling, it remains insufficient to build a universal quantitative model linking mechanical inputs to cell fate prediction. Therefore, the rheostat model is proposed here as a testable conceptual framework, predicting that distinct combinations of mechanical parameters may bias Piezo1 signaling toward adaptive osteogenesis, metabolic compensation, inflammatory activation, or cell death. By organizing Piezo1-mediated signaling into an integrated network, this review provides a refined perspective for understanding skeletal mechanical adaptation and the therapeutic potential of Piezo1-targeted “mechano-mimetics”.

## 2 Structural basis and activation mechanisms of Piezo1

### 2.1 Three-dimensional structural architecture

Piezo1 does not function as a monomer but rather assembles into a massive homotrimeric complex. Each subunit comprises over 2,500 amino acid residues and up to 38 transmembrane helices (TMs), resulting in a total molecular weight of approximately 900 kDa, making it one of the largest known ion channels (Saotome et al., 2018; Ge et al., 2015). This massive architecture constitutes the fundamental framework for mechanosensation and transduction. The overall structure exhibits a unique three-bladed propeller-like conformation. Its core functional domains include the central ion-conducting pore and the peripheral “blades”. The pore is formed by the pore-lining transmembrane helices within the C-terminal pore module, whereas the C-terminal extracellular domain flanks the pore and contributes to pore architecture and gating rather than directly constituting the ion-conducting pathway. The peripheral blades, formed by repetitive units of four-transmembrane helical bundles, serve as the major mechanosensing modules. These large, curved blades are considered the primary mechanosensing modules (Saotome et al., 2018; Zhao et al., 2019). Structurally connecting the peripheral blades to the central pore are the intracellular “Beams” and “Anchors” (Saotome et al., 2018), which act as molecular levers to transmit conformational changes from the blades to the pore over a long distance. Additionally, the Piezo1 structure features a unique “cap” domain positioned above the central pore, functioning as the “valve” for channel gating. The core of its activation mechanism is described by the “Force-from-lipids” gating model. This model posits that the massive peripheral blades of Piezo1 are tightly coupled to the lipid bilayer, deforming the surrounding membrane into a “nanodome” shape. When the cell membrane undergoes stretching or compression, changes in membrane tension or curvature act directly on the blades, inducing conformational distortion and rotation. These conformational rearrangements are mechanically transmitted via the intracellular beams and anchors to the central pore and cap domain, ultimately triggering channel opening and converting physical forces on the membrane into ionic currents (Vasileva and Chubinskiy-Nadezhdin, 2023; Syeda et al., 2016). Cryo-electron microscopy (Cryo-EM) analysis has confirmed significant structural distinctions between the closed and open states, providing further structural evidence for this mechanogating mechanism (Verkest et al., 2025).

### 2.2 Diversity of activation modes: the foundation of the “rheostat”

Piezo1 is a versatile mechanosensor capable of responding to a broad spectrum of physical and chemical stimuli (Lacroix and Wijerathne, 2025). It not only detects mechanical inputs but also functions as a highly specialized mechanotransducer that precisely decodes the physical properties of diverse stimuli, triggering signaling cascades involved in vascular vasodilation, remodeling, and angiogenesis (Chi et al., 2022; Kang et al., 2019). This capability serves as the structural and functional basis for its role as a “Mechanical Signal Rheostat” Membrane tension represents the most direct and fundamental activation mode. Membrane stretching or compression—whether induced by cell swelling, micropipette aspiration, or changes in extracellular matrix (ECM) stiffness (where stiffer matrices increase membrane stretch)—can directly activate Piezo1 (Smith et al., 2025; Javed et al., 2025). In bone tissue, cellular deformation is the primary mode of load sensing. For instance, osteocytes residing within the lacunar-canalicular network (LCN) sense fluid shear stress (FSS) generated by interstitial fluid flow via their dendritic processes; this has been confirmed as a critical physiological stimulus for Piezo1 activation in osteocytes (Li M. et al., 2024; Zhan et al., 2024). Similarly, in articular cartilage, the compressive forces experienced by chondrocytes constitute a vital mechanical input, with Piezo1 playing a key regulatory role (Du J. et al., 2025). Physical modalities such as low-frequency ultrasound and vibration can also activate Piezo1 by generating local pressure or shear forces, thereby altering downstream pathways to elicit distinct biological outcomes (Jiang et al., 2025; Zeng et al., 2025). Pharmacological agonists and inhibitors provide useful experimental tools for investigating Piezo1 function. (Xiao, 2020; Thien et al., 2024). These agonists and inhibitors provide essential pharmacological tools for simulating or modulating mechanical stimulation both *in vitro* and *in vivo*. Crucially, Piezo1 characteristics are heavily dependent on the lipid microenvironment—a combination of the plasma membrane, cytoskeleton, and ECM. The lipid composition of the membrane exerts a significant influence on Piezo1 activity (Vasileva and Chubinskiy-Nadezhdin, 2023). For example, *in vitro* alteration of cholesterol concentration can inhibit Piezo1 activity, while phosphatidylinositol 4,5-bisphosphate (PIP2) and fatty acids may modulate activity by altering membrane tension, directly interacting with the channel, or modifying membrane structural properties (Chong et al., 2021). In addition to the “force-from-lipids” model, the “force-from-filaments” theory provides a complementary mechanism for Piezo1 activation. According to this view, mechanical force may be transmitted to Piezo channels through cytoskeletal or extracellular matrix-associated filamentous structures, including actin filaments, cadherin-associated complexes, integrin-based adhesions, and matrix components. Thus, Piezo1 mechanogating in musculoskeletal cells may depend not only on lipid bilayer tension and membrane curvature but also on cytoskeletal prestress and matrix-derived force transmission.

The response of Piezo1 to mechanical stimuli is not a binary “on-off” switch but is highly dependent on the characteristics of the input signal; this differential responsiveness is the core essence of the “Mechanical Signal Rheostat”. While Piezo1 is sensitive to various inputs like stretch, compression, and shear stress, its activation kinetics and downstream signaling biases may differ (Savadipour et al., 2023b). Furthermore, Piezo1 activation typically exhibits a threshold effect and follows a dose-dependent manner within a specific intensity range, reaching a plateau upon saturation. Notably, static loading versus dynamic oscillatory loading significantly impacts the activation pattern of Piezo1 and the duration of the resulting Ca^2+^ signals (Liu et al., 2020). For instance, low-frequency dynamic loading may be more effective than static loading in promoting osteogenesis. Transient versus sustained stimuli elicit distinct patterns of Ca^2+^ transients, thereby influencing the selection and activation intensity of downstream signaling pathways (Yang X. et al., 2022; Zhao et al., 2018). Through Piezo1, these varying mechanical parameters are “encoded” into Ca^2+^ signals with specific kinetic signatures, which then differentially regulate the activation ratios and patterns of multidimensional downstream signaling axes, ultimately determining cell fate.

### 2.3 Ion permeability and electrophysiological properties

The opening of the Piezo1 channel primarily mediates the transmembrane flow of cations, and its electrophysiological properties are key to understanding its biological function. Piezo1 is classified as a non-selective cation channel, permeable to monovalent ions (Na^+^, K^+^) and divalent ions (Ca^2+^, Mg^2+^) (Hirata et al., 2023). However, under physiological conditions, due to the steep electrochemical gradient and a slight preference for Ca^2+^, the transient elevation of intracellular Ca^2+^ concentration is the most significant biological consequence of its opening (Hill et al., 2022). Using cell-attached patch-clamp techniques and pressure-clamp stimulation, studies have demonstrated that Piezo1 functions as an independent mechanosensor regardless of expression density. Its pressure sensitivity and open probability are not significantly affected by channel density, a characteristic essential for the uniform transduction of mechanical signals across the cell (Lewis and Grandl, 2021; Murciano et al., 2023). Electrophysiologically, Piezo1 exhibits rapid activation and inactivation kinetics upon mechanical stimulation. Particularly under sustained stimulation, the channel current decays rapidly. This rapid inactivation is critical for the “rheostat” function, enabling cells to distinguish between transient and sustained mechanical stimuli and to make refined adaptive responses to dynamic changes (Liu S. et al., 2025). Research has revealed a molecular mechanism involving a physical “plug-and-latch” system that finely gates the intracellular ion permeation pathway. Researchers also identified a splice variant (Piezo1.1) lacking the “plug” structure; this variant exhibits larger single-channel conductance and higher mechanosensitivity, directly proving the critical role of this structural element in controlling channel opening and inactivation (Lewis and Grandl, 2021; Geng et al., 2020). This rapid inactivation mechanism confers an adaptive capability to Piezo1, preventing sustained overload of intracellular Ca^2+^ signals and avoiding potential cytotoxicity. Such adaptation also allows Piezo1 to reset quickly to a sensitive state after stimulus removal, readying the cell for subsequent mechanical events (Liu et al., 2025b).

### 2.4 Spatiotemporal expression and regulatory dynamics in bone-related cells

In bone-related cells, Piezo1 expression and activity are subject to multidimensional and finely tuned dynamic regulation, thereby precisely calibrating its function as a mechanical “rheostat”. Beyond its structural gating properties, the function of Piezo1 in musculoskeletal tissues is critically determined by its expression level and regulatory state. Mechanical loading itself can regulate Piezo1 expression, thereby establishing a feed-forward mechanism that adjusts cellular mechanosensitivity according to the local mechanical environment. In osteoblast-lineage cells and osteocytes, appropriate mechanical cues generally maintain or enhance Piezo1 expression, enabling these cells to respond efficiently to fluid shear stress, matrix stiffness, and tissue-level deformation. This process is usually associated with the upregulation of key osteogenic factors such as bone morphogenetic protein 2 (BMP2) (Huang et al., 2025; Sun et al., 2019). In contrast, aging, unloading, oxidative stress, and inflammatory stress may reduce Piezo1 expression or impair its mechanosensitive responsiveness, leading to weakened skeletal adaptation and impaired bone formation (Lei et al., 2024). At the transcriptional level, Piezo1 expression appears to be regulated by mechanosensitive transcriptional programs, including YAP/TAZ-related feedback regulation and other mechanically responsive transcription factors (Wu Z. et al., 2025). This transcriptional control enables bone-resident cells to adjust their mechanosensing capacity according to changing mechanical demands. Activation of Piezo1 promotes calcium signaling and intracellular mechanotransduction pathways, thereby enhancing the proliferation, migration, and differentiation of BMSCs and accelerating the repair of bone defects. This positive feedback loop ensures that, upon sensing osteogenic mechanical signals, BMSCs can enhance their own sensitivity to force and thus differentiate more efficiently toward the osteogenic lineage (Xu C. et al., 2025). For osteoblasts, Piezo1 enables these cells to efficiently sense bone surface tension and execute bone formation. Mice with osteoblast-lineage-specific deletion of Piezo1 exhibit severe skeletal developmental defects, including impaired bone formation, markedly reduced bone mass, decreased bone strength, and even spontaneous fractures. These phenotypes provide strong evidence that Piezo1 is required for osteoblasts to sense and respond to mechanical loading (Zhang et al., 2025a; Wang et al., 2020). At the post-transcriptional level, non-coding RNAs and RNA stability mechanisms may further fine-tune Piezo1 abundance, although their precise roles in skeletal tissues remain insufficiently defined. Piezo1 can regulate osteoblast-specific gene expression by modulating downstream pathways such as ERK and p38 and by interacting with Hippo signaling. Piezo1 can also regulate β-catenin activation and subsequently activate Runx2, thereby controlling osteoblast gene expression. In addition, Piezo1 activates NFATc1 and YAP through calcium signaling, after which NFATc1 and YAP translocate into the nucleus to regulate osteoblast differentiation (Zhu et al., 2024; Zhang T. et al., 2025). As Runx2 is a core transcription factor for osteogenic differentiation, fluid shear stress can induce Runx2 expression by upregulating Piezo1, whereas inhibition of Piezo1 blocks the mechanically induced upregulation of Runx2 (Song et al., 2020). Piezo1 promotes Wnt1 expression through YAP/TAZ activation, which not only enhances bone formation but also reduces bone resorption (Xu et al., 2021). At the protein level, post-translational modifications, such as phosphorylation and changes in the lipid microenvironment, may alter Piezo1 sensitivity, inactivation kinetics, and membrane availability without necessarily changing total Piezo1 expression (Zhang T. et al., 2024). In contrast to the osteogenic lineage, the regulatory mechanism of Piezo1 in osteoclasts—the bone-resorbing cells—functions as a negative feedback brake. Activation of Piezo1 in osteoclast precursors negatively regulates NFATc1 expression and inhibits its nuclear translocation, thereby suppressing differentiation, reducing osteoclast formation, and limiting resorption pit formation (Shindo et al., 2025; Wang et al., 2020; Shindo et al., 2024). This suggests that in the osteoclast lineage, Piezo1 is subjected to distinct transcriptional control (potentially inverse regulation by factors like NFATc1) and post-translational modifications to prevent excessive activation and subsequent bone loss. Articular cartilage is a tissue subjected to immense mechanical loading. Chondrocytes, the sole cell type responsible for ECM maintenance, rely on Piezo1 for homeostasis. Under physiological loading ranges, moderate Piezo1 activation is essential for maintaining chondrocyte health and ECM balance (Yuan et al., 2025). By regulating basal intracellular Ca^2+^ levels, it supports normal metabolism and matrix synthesis (Lee et al., 2014). Piezo1 is also a critical regulator of endochondral ossification; its expression is physiologically necessary for postnatal trabecular bone formation beneath the growth plate (Brylka et al., 2024). However, under pathological conditions, the narrative shifts. Specific deletion of Piezo1 in aging chondrocytes upregulates Apolipoprotein E (ApoE) in hypertrophic chondrocytes, delaying the transition from cartilage to bone (Jia S. et al., 2024). Conversely, excessive mechanical stress induces mitochondrial DNA release via Piezo1, triggering the cGAS-STING pathway and exacerbating cartilage degeneration (Sun L. et al., 2025). Furthermore, Piezo1 deficiency leads to mitochondrial dysfunction (reduced membrane potential and ATP synthesis), hindering transdifferentiation into osteoblasts (Zhang T. et al., 2025). Mechanically induced Piezo1 activation also promotes the Senescence-Associated Secretory Phenotype (SASP), particularly IL-6 and IL-1β, via p38 MAPK and NF-κB pathways, revealing a mechanistic link between aberrant mechanical stress and Piezo1-mediated chondrocyte senescence (Liu Y. et al., 2023). Piezo1 is functionally expressed in tenocytes, serving as a key modulator of tendon biomechanics. A gain-of-function Piezo1 variant (E756del), found at high frequency in African descent populations, has been linked to superior athletic performance (specifically sprinting). Biomechanical analysis reveals that tendons in mice carrying this variant exhibit higher compliance and elastic energy storage capacity, correlating with enhanced jumping ability (Nakamichi et al., 2022). In humans, however, this variant has also been associated with increased patellar tendon stiffness (Götschi et al., 2023). In skeletal muscle, regeneration relies on Muscle Satellite Cells (MuSCs). Interestingly, Piezo1 is highly enriched in quiescent and activated MuSCs but is barely detectable in mature myofibers, indicating a specialized role in the stem cell stage of regeneration. Genetic ablation of Piezo1 in MuSCs severely delays muscle repair. Mechanistically, Piezo1 plays a precise regulatory role during mitosis by localizing to the cleavage furrow and modulating RhoA-GTPase activity to ensure successful cytokinesis and stem cell expansion (Hirano et al., 2023). Furthermore, during the fusion of MuSCs into myotubes, Piezo1-mediated Ca^2+^ influx is critical; its inhibition leads to abnormal myotube morphology due to defects in actomyosin assembly, identifying Piezo1 as a determinant of myofiber morphogenesis (Lei et al., 2024). Although our mainly focuses on bone-resident cells, non-bone stromal and immune cells also contribute substantially to skeletal homeostasis and disease. Macrophages in the bone marrow microenvironment respond to mechanical and inflammatory cues and can influence angiogenesis, osteogenesis, and osteoclastogenesis. Piezo1 activation in bone marrow macrophages has been reported to promote VEGF-A production through the CaN/NFAT/HIF-1α pathway, thereby supporting vascular regeneration and bone marrow niche repair (Zhang X. et al., 2022). In addition, Piezo1-mediated mechanotransduction in M2-like macrophages may enhance bone formation through the secretion and activation of transforming growth factor-β1 (TGF-β1) (Cai et al., 2023). Fibroblasts and myofibroblasts are also relevant to bone and joint health because they regulate extracellular matrix deposition, tissue stiffness, periosteal and synovial remodeling, and fibrotic repair. In these cells, Piezo1 may couple matrix stiffness and cytoskeletal tension to calcium influx, focal adhesion signaling, YAP/TAZ activation, and collagen remodeling (Lin Y. et al., 2025). Therefore, Piezo1 should be considered not only as a mechanoregulator of classical skeletal cells but also as a mediator of immune–stromal crosstalk within the skeletal microenvironment. The regulation of Piezo1 should be regarded as a multilayered process involving transcriptional control, post-transcriptional fine-tuning, protein-level modulation, and membrane-dependent regulation. Together, these mechanisms determine how osteoblasts, osteocytes, chondrocytes, osteoclast precursors, and skeletal progenitor cells calibrate their responses to mechanical stimuli (Table 1).

**TABLE 1:** Expression and function of Piezo1 in key musculoskeletal and bone-associated.

Cell type	Primary physiological function	Role in pathophysiology	Key downstream pathways
Osteoblasts/Osteocytes	Senses mechanical loading to drive osteogenesis and bone formation; inhibits Sclerostin expression	Downregulation leads to disuse and senile osteoporosis	YAP/TAZ, Wnt/β-catenin, Akt
Osteoclasts	Inhibits differentiation and bone resorption activity under mechanical loading	Loss of mechanical signaling leads to unchecked osteoclast activity and exacerbated bone resorption	PP2A-Akt
Chondrocytes	Maintains extracellular matrix (ECM) homeostasis under physiological loading	Hyperactivation under injurious loading or inflammation drives Osteoarthritis (OA) progression	Calcineurin-NFAT, ER Stress
Tenocytes	Regulates tendon stiffness and elastic energy storage; influences athletic performance	Gain-of-function variant correlates with enhanced athletic performance	Collagen cross-linking pathways
Muscle satellite cells (MuSCs)	Promotes activation, proliferation, and differentiation of quiescent MuSCs; critical for muscle regeneration	Deletion or dysfunction delays post-injury repair and promotes cellular senescence	RhoA, Actomyosin assembly
Macrophages	Regulate angiogenesis, osteogenesis, and immune remodeling in the bone marrow microenvironment	Aberrant activation may amplify inflammatory remodeling and osteoclastogenic signals	CaN/NFAT/HIF-1α/VEGF-A; TGF-β1
Fibroblasts/myofibroblasts	Regulate ECM deposition, tissue stiffness, periosteal/synovial remodeling, and repair	Excessive activation contributes to fibrosis and pathological matrix remodeling	Ca^2+^ influx, focal adhesion signaling, YAP/TAZ, collagen remodeling

## 3 The Piezo1 signaling rheostat: five tunable axes and their crosstalk

In living skeletal cells, a given mechanical stimulus usually engages multiple Piezo1-dependent modules simultaneously. For example, Piezo1-mediated calcium influx can activate calcineurin/NFAT-dependent transcription, promote CaMKII signaling, reshape actin cytoskeletal tension through RhoA/ROCK, facilitate YAP/TAZ nuclear translocation, and alter mitochondrial calcium uptake. These events are temporally coupled and mutually reinforcing. Therefore, the “Piezo1 signaling rheostat” should be understood as an integrated signaling network in which calcium, cytoskeletal tension, metabolic state, inflammatory stress, and transcriptional control converge to determine cell fate.

### 3.1 The ionic cascade axis

As the premier mechanosensitive ion channel in bone tissue, the Piezo1 ionic cascade axis represents the vanguard of mechanotransduction, converting physical forces into biochemical signals. This process follows a precise sequence: physical force (millisecond response) → Piezo1 opening→ patterned Ca^2+^ influx →activation of specific decoders → promotion of osteogenesis (Gao et al., 2022; Wang et al., 2025b). This axis is characterized not only by its rapidity but also by its highly “digital” nature. It is the amplitude, duration, frequency, and spatial distribution (spatiotemporal dynamics) of Ca^2+^ signals—rather than mere cytosolic concentration—that dictate downstream cellular fate, ensuring the precise biological translation of mechanical stimuli. Mechanical inputs such as FSS, matrix stiffness, compression, or vibration directly deform the Piezo1 trimer. The channel’s open probability correlates positively with force intensity, thereby determining the magnitude of Ca^2+^ flux (Xu et al., 2021; Jia B. Z. et al., 2025). A defining electrophysiological feature of Piezo1 is its rapid inactivation kinetics, with a half-inactivation time constant of approximately 10–20 ms (Wu et al., 2017). This property renders the channel highly sensitive to transient, dynamic mechanical stimuli while preventing sustained activation under static loads, effectively functioning as a “high-pass filter” for mechanical noise. In the Osteogenic Lineage (Osteoblasts, Osteocytes, and BMSCs):Physiological FSS induces Piezo1-mediated localized Ca^2+^ entry, which triggers a canonical downstream cascade. These Ca^2+^ transients activate Calcineurin and CaMKII, acting as signal decoders. This activation drives the dephosphorylation and nuclear translocation of NFATc1, YAP1, and β-catenin, facilitating the formation of transcriptional complexes that upregulate osteogenic genes such as Runx2, Osterix, and Col1a1 ^90^. Furthermore, studies using cyclic tension have demonstrated that Piezo1 upregulation accompanies Ca^2+^ influx, promoting the expression and phosphorylation of CaMKII. Concurrently, YAP and β-catenin signaling are enhanced, suggesting that the Piezo1-Ca^2+^ axis may orchestrate mechanically induced osteogenesis via the CaMKII pathway (Du Y. et al., 2025). This classical Ca^2+^-dependent cascade dominates the osteogenic lineage, enhancing Type II/IX collagen secretion via the YAP/TAZ axis and upregulating key osteogenic factors (NFATc1, Wnt3a, β-catenin), thereby indirectly inhibiting osteoclast differentiation (Wu Z. et al., 2025; Wang et al., 2025b; Zhao T. et al., 2025). In a striking contrast, Piezo1 activation in osteoclast precursors switches to a unique Ca^2+^-independent cascade (or non-canonical pathway). Here, Piezo1 directly recruits and activates PP2A, leading to Akt dephosphorylation. This event subsequently downregulates NFATc1 expression and inhibits its nuclear translocation, thereby suppressing RANKL-induced expression of TRAP, CTSK, and DC-STAMP (Shindo et al., 2025; Shindo et al., 2024). This mechanism functions as an intrinsic negative feedback “brake” to prevent excessive bone resorption. This precise lineage-specific dichotomy—Ca^2+^-dependent activation of NFATc1 in osteoblasts versus Ca^2+^-independent inhibition of NFATc1 in osteoclasts—highlights the sophistication of the Piezo1 rheostat. It avoids conflict with the RANKL-ITAM signaling pathway in osteoclasts, ensuring the maintenance of skeletal homeostasis under mechanical loading (Table 2).

**TABLE 2:** Lineage-specific comparison of the Piezo1-mediated ionic cascade axis.

Network dimension	Osteogenic lineage	Osteoclast lineage	Integrated significance
Triggering signal	Mechanical loading → Piezo1-mediated Ca^2+^ influx (Gao et al., 2022; Wang et al., 2025b)	Mechanical loading → Piezo1-associated PP2A activation (Shindo et al., 2024)	Lineage-specific decoding of mechanical inputs
Core mediators	Calcineurin, CaMKII, NFAT, YAP/TAZ, β-catenin (Zhou et al., 2020; Du et al., 2025b; Zhao et al., 2025a)	PP2A–Akt–NFATc1 axis (Shindo et al., 2025; Shindo et al., 2024)	Divergent regulation of NFAT-related programs
Downstream output	Runx2, Osx, Col1a1 and osteogenesis ↑ (Zhou et al., 2020)	TRAP, CTSK, DC-STAMP and osteoclastogenesis ↓ (Shindo et al., 2025; Shindo et al., 2024)	Bone formation favored while resorption is restrained
Pathological relevance	Impaired Ca^2+^ signaling contributes to defective osteogenesis (Shindo et al., 2025)	Loss of inhibitory signaling may enhance resorption (Du et al., 2025b)	Disrupted balance contributes to bone loss
Therapeutic implication	Piezo1 activation may enhance osteogenic signaling	Piezo1-related pathways may restrain osteoclast differentiation	Potential mechano-mimetic strategy

Within the osteogenic lineage (osteocytes, osteoblasts, and BMSCs), the ionic cascade axis orchestrates a multi-branched network, where distinct downstream decoders exhibit differential sensitivity to specific Ca^2+^ patterns (Table 3).

**TABLE 3:** Differential decoding of Piezo1-mediated Ca^2+^ patterns in the osteogenic lineage.

Decoder branch	Ca^2+^ pattern preference	Key mechanism	Downstream output (osteogenic)	Refs
Calcineurin-NFAT Axis	Sustained medium-to-high amplitude plateau	Calcineurin/CaM-dependent dephosphorylation of NFATc1 →Nuclear translocation + Complex formation with YAP/β-catenin	↑*Runx2*, *Osx*, *Ocn*; Assembly of core osteogenic transcriptional complexes	Zhou et al. (2020)
CaM-CaMKII/IV Axis	High-frequency oscillations	Autophosphorylation (“Memory effect”) →p-CREB activation	↑*c-fos*, Survival signals; Synergy with β-catenin	Wang et al. (2025a), Du et al. (2025b)
PKC-ERK/MAPK Axis	Transient high-amplitude spikes or oscillations + PLC-DAG	PKC →Raf-MEK-ERK Cascade	↑Proliferation, *Cyclin D1*; Biasing BMSC lineage commitment toward osteogenesis	Wang et al. (2025c), Xie et al. (2024)
YAP/TAZ Integrated branch	Localized microdomains + F-actin rearrangement	Ca^2+^-dependent actin polymerization → YAP nuclear translocation	↑*Col1a1*, *Cyr61*; Collagen secretion and pathway crosstalk	Zhao et al. (2025a), Xu et al. (2025b)
CICR Amplification loop	Any initial influx	RyR/IP3R Positive feedback + Mitochondrial buffering	Whole-cell wave propagation; global amplification of all branches	Pach et al. (2000), Xue et al. (2025), Bloch et al. (2025)

### 3.2 The cytoskeletal-nuclear mechanotransduction axis

**FIGURE 1:**
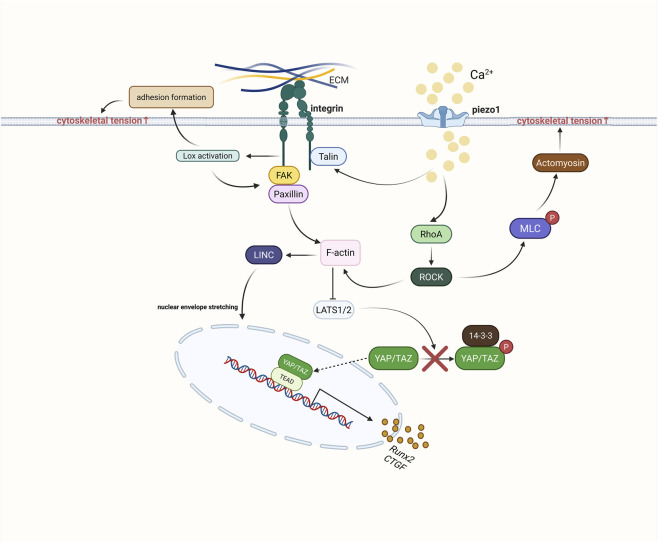
Schematic representation of the Piezo1-mediated cytoskeletal-nuclear mechanotransduction axis.

Distinct from the ionic axis which prioritizes “chemical signaling”, the Cytoskeletal-Nuclear Mechanotransduction Axis represents a “physical signal transmission chain” that propagates forces from the extracellular matrix (ECM) directly to the nucleus to drive transcriptional programs. In bone-related cells, the activation of Piezo1 not only triggers Ca^2+^ influx but is also tightly coupled with cytoskeletal tension and nuclear mechanical deformation, thereby regulating the activity of mechanosensitive transcription factors like YAP/TAZ to dictate osteogenic fate and homeostasis. The focal adhesion Nexus Piezo1 activation induces local membrane deformation and Ca^2+^ influx, a process that synergizes with integrin-mediated adhesion signaling (Cheng et al., 2023; Wang J. et al., 2025). Piezo1 activation is closely coupled with integrin-mediated adhesion signaling. When mechanical force activates Piezo1, the resulting Ca^2+^ influx can promote talin activation and strengthen integrin–extracellular matrix (ECM) engagement. This process facilitates the recruitment of focal adhesion kinase (FAK) and paxillin, thereby promoting focal adhesion maturation and actin cytoskeletal remodeling. Mature focal adhesions anchor actin stress fibers and physically connect the ECM to the intracellular cytoskeleton, allowing Piezo1-dependent calcium signals and matrix-derived mechanical cues to be integrated at the cytoskeletal level and providing the structural basis for subsequent force transmission (Cheng et al., 2023; Liang et al., 2024; Liu X. et al., 2023; Yang M. et al., 2025; Yao et al., 2022). This focal adhesion–actin network further activates the RhoA/ROCK pathway. ROCK phosphorylates myosin light chain (MLC), increases actomyosin contractility, and enhances cytoskeletal prestress (Zhao T. et al., 2025; Masalmeh et al., 2025). In osteoblast-related studies, inhibition of ROCK activity reduced MLC phosphorylation and was thought to promote human osteoblast migration by reorganizing the actin cytoskeleton and regulating myosin activity (Zhang et al., 2011). This tension-regulatory mechanism is highly associated with YAP/TAZ nuclear localization. Increased actin tension promotes YAP/TAZ activation by reducing LATS1/2-mediated inhibitory phosphorylation and facilitating force transmission to the nucleus through the LINC complex. Through this process, Piezo1 links membrane-level mechanosensing to focal adhesion remodeling, cytoskeletal tension, nuclear mechanotransduction, and transcriptional regulation (Zhou et al., 2025a; Zheng et al., 2022; Aragona et al., 2013). Mechanistically, enhanced F-actin polymerization and stretching upregulate the expression of the Linker of Nucleoskeleton and Cytoskeleton (LINC) complex. This complex physically pulls on the nuclear envelope, expanding the Nuclear Pore Complexes (NPCs) and establishing physical continuity between the cytoskeleton and the nucleus (Meng et al., 2024). Under high cytoskeletal tension, Mechanical signals driven by F-actin are transmitted via the RhoA–ROCK axis to the Hippo core kinase module, blocking the activation of LATS1/2. Reduced LATS1/2 activity prevents the inhibitory phosphorylation of YAP/TAZ, maintaining them in an unphosphorylated, active conformation (Sato et al., 2023; Zhou et al., 2022). Since 14-3-3 proteins specifically bind phosphorylated YAP/TAZ, this dephosphorylation allows YAP/TAZ to escape 14-3-3-mediated cytoplasmic retention, gaining “transport freedom” (Kofler et al., 2018). High tension mechanically stretches the nuclear skeleton via the LINC complex, causing the physical expansion of NPCs, which significantly increases the rate of macromolecular nuclear transport (Meng et al., 2024). Through this dual mechanism, YAP/TAZ efficiently enters the nucleus and binds to the TEAD family transcription factors to initiate mechanotransduction-dependent gene programs. This includes the upregulation of Runx2 and CTGF, which directly promotes differentiation and matrix production in osteoblasts (Nijsure et al., 2025; Crespo-Enriquez et al., 2019; Yang et al., 2018). This process constitutes the core nexus of the Piezo1–Cytoskeleton–Nuclear Axis, converting mechanical stimuli into transcriptional outputs. Specifically, the mechanical regulation of nuclear envelope tension and NPC conformation to facilitate macromolecular import represents a current research hotspot. In osteoblasts, Piezo1 activation has been explicitly observed to promote YAP/TAZ nuclear localization and enhance osteogenic programs, tightly bridging the ionic pathway with the nuclear mechanotransduction axis (Liu C. et al., 2025). Collectively, Piezo1 realizes a cross-scale signal transduction—from “single-point mechanical stimuli” to “gene expression reprogramming”—through a progressive amplification system: Membrane Deformation → focal Adhesion Signaling →RhoA/ROCK→ LINC →YAP/TAZ. This axis serves as a critical mechanism for skeletal mechanical adaptation and bone mass maintenance (Figure 1).

### 3.3 The metabolic reprogramming axis

**FIGURE 2:**
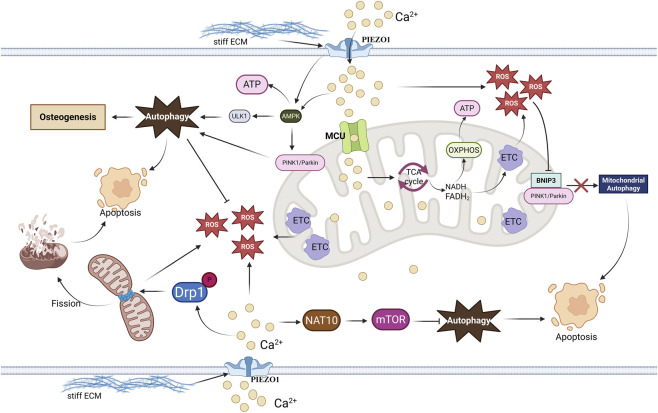
The Piezo1-mediated metabolic reprogramming axis: coupling mechanics to energy and fate.

Mechanosensation is an inherently energy-intensive process. Bone-related cells require substantial energy expenditure to support cytoskeletal remodeling, ion pump operation, and *de novo* protein synthesis during their response to mechanical loads. Piezo1 ingeniously couples mechanical signals with cellular energy metabolism by regulating mitochondrial function and key metabolic switches, thereby ensuring energy supply under physiological conditions or precipitating metabolic collapse under pathological states (Zhang T. et al., 2025). The cytosolic Ca^2+^ influx triggered by Piezo1 acts not merely as a rapid ionic signal but as a primary trigger for metabolic reprogramming. The Mitochondrial Calcium Uniporter (MCU) located on the inner mitochondrial membrane rapidly sequesters and “buffers” the elevated cytosolic Ca^2+^ (Modesti et al., 2021; Patel et al., 2022). This process shapes the spatiotemporal pattern of cytosolic Ca^2+^ signals. More crucially, the entry of Ca^2+^ into the mitochondrial matrix allosterically regulates Pyruvate Dehydrogenase and key enzymes of the Tricarboxylic Acid (TCA) cycle (such as Isocitrate Dehydrogenase and α-Ketoglutarate Dehydrogenase). This leads to increased TCA flux (Mammucari, 2024) and elevated production of NADH and FADH_2_, which in turn drives the Electron Transport Chain (ETC) and Oxidative Phosphorylation (OXPHOS). This efficient synthesis of ATP provides the necessary “chemical energy” to fuel the cell’s “mechanical work” (Li X. et al., 2022). However, enhanced ETC activity inevitably generates Reactive Oxygen Species (ROS) as byproducts. While physiological ROS can act as signaling molecules, hyperactivation of Piezo1 leads to mitochondrial Ca^2+^ overload and a deleterious ROS burst. This further suppresses the expression of PINK1, Parkin, and BNIP3, exacerbating defective mitophagy under mechanical stress and inducing apoptosis, which aggravates cartilage damage (Yu et al., 2025a). AMPK and mTOR serve as the central governors of cellular energy status. Piezo1 significantly activates cellular AMPK signaling, which enhances the PINK1/Parkin pathway, promotes mitophagy, improves mitochondrial function, and reduces oxidative stress (Yang Y. et al., 2025). In bone tissue, Piezo1-mediated Ca^2+^ influx acts as a second messenger to activate AMPK and ULK1, maintaining a moderate level of autophagy that favors osteoblast differentiation (Wu et al., 2023). Conversely, the intensity of autophagy dictates distinct cellular outcomes. In the Cartilage Endplate (CEP)—a critical component of the spinal functional unit—compressive loading activates Piezo1, increasing Ca^2+^ influx and upregulating the target gene NAT10. Elevated NAT10 levels enhance mTOR stability, thereby inhibiting autophagy and promoting apoptosis (Sun H. et al., 2025). This highlights a context-dependent regulation where Piezo1 can tip the balance between survival (AMPK-mediated autophagy) and death (mTOR-mediated inhibition). Furthermore, Piezo1 dictates lineage fate by modulating mitochondrial dynamics (fission vs. fusion). During Intervertebral Disc Degeneration (IDD), increased matrix stiffness in the Nucleus Pulposus (NP) promotes Piezo1 activation, leading to Drp1 phosphorylation, mitochondrial fission, and subsequent NP cell apoptosis (Ke et al., 2023) (Figure 2). The morphological state of mitochondria is intimately linked to metabolic phenotype: fissioned mitochondria are often associated with high glycolysis and osteogenic differentiation, whereas fused mitochondria correlate with high OXPHOS and adipogenic lineage commitment (Huang et al., 2025; Liu et al., 2025d). The Piezo1-mediated balance of intracellular Ca^2+^ and ROS serves as a critical regulator of the fission protein Drp1 and the fusion protein Mitofusin (Mfn). This represents a novel and pivotal metabolic mechanism by which Piezo1 governs BMSC fate.

### 3.4 The inflammatory-stress response axis

While Piezo1 functions as a homeostatic regulator under physiological conditions, it acts as a “double-edged sword”. When mechanical stimuli exceed physiological thresholds, Piezo1 activation shifts from adaptive to injurious, initiating inflammatory and cellular stress programs. This transition is particularly prominent in degenerative diseases such as Osteoarthritis (OA), where Piezo1 transduces aberrant mechanical forces into inflammatory signals, amplifying tissue damage (Zhou et al., 2025b). The core mechanisms orchestrating this shift involve a feedback loop of calcium overload-induced Endoplasmic Reticulum (ER) stress, canonical NF-κB activation, NLRP3 inflammasome assembly, and the triggering of diverse cell death modalities, all contributing to chronic inflammation and the disruption of skeletal homeostasis (Yu et al., 2025b). Piezo1 plays diverse roles in physiology, but its pathological hyperactivation leads to sustained cytosolic Ca^2+^ elevation. This influx overwhelms the Sarco/Endoplasmic Reticulum Ca^2+^-ATPase (SERCA)—the primary pump responsible for sequestering cytosolic Ca^2+^ back into the ER—thereby disrupting ER calcium homeostasis and triggering the accumulation of unfolded proteins. This initiates the Unfolded Protein Response (UPR), mediated by three transmembrane sensors: IRE1, ATF6, and PERK (Zhao J.-X. et al., 2025; Zhang et al., 2025c). Initially, the UPR aims to restore ER quality control and relieve cellular stress (Wiseman et al., 2022). However, unmitigated mechanical stress shifts the UPR toward a maladaptive terminal phase. Activation of the pro-apoptotic transcription factor CHOP via the PERK pathway generates Reactive Oxygen Species (ROS) and activates the NLRP3 inflammasome, mediating sterile inflammation and apoptosis (Zha et al., 2024). In knee OA models, aberrant stress correlates with upregulated UPR markers (XBP1s, ATF6) via Piezo1, whereas Piezo1 inhibition preserves cartilage viability by dampening these pathways (Song et al., 2025). Beyond the ER, Piezo1 mediates the release of mitochondrial DNA (mtDNA) into the cytosol, activating the cGAS-STING pathway, which further exacerbates inflammation and cartilage damage (Sun L. et al., 2025). Furthermore, Piezo1 serves as a master switch for the NF-κB pathway. Ca^2+^ influx promotes IKK-mediated degradation of IκB, unleashing NF-κB to drive the transcription of pro-inflammatory cytokines (IL-1β, IL-6, TNF-α) and catabolic enzymes (MMP13, COX-2) (Du et al., 2025c). In osteoclasts, this enhances sensitivity to RANKL, accelerating bone resorption. The NLRP3 inflammasome acts as the “storm center” of this cascade. Piezo1 priming via NF-κB and activation via ROS upregulation promotes the secretion of IL-1β and IL-18, fueling synovial inflammation and fibrosis (Yu et al., 2025b; Zhou P. et al., 2025). This mechanism is also evident in Intervertebral Disc Degeneration (IVDD), where Piezo1 drives NLRP3-mediated inflammation in nucleus pulposus cells (Sun et al., 2020). Piezo1 activation determines the specific mode of cell death in a context-dependent manner. In Vertebral Growth Plate Disorder (VGPD), Piezo1 upregulation disrupts GPX4 signaling, amplifying ferroptosis in chondrocytes and accelerating pathological ossification (Chen F. et al., 2025). In periodontitis, excessive Ca^2+^ influx triggers Gasdermin D (GSDMD) cleavage and membrane pore formation, leading to pyroptosis and impairing the osteogenic differentiation of periodontal ligament stem cells (Chen P. et al., 2025). In IVDD, aberrant activation under mechanical loading induces apoptosis in Annulus Fibrosus Cells (AFCs) via the Calpain2/BAX/Caspase3 axis, compromising disc structural integrity (Liu C. et al., 2023). Finally, it is crucial to recognize the temporal duality of Piezo1-mediated inflammation. Acute activation induces transient “adaptive inflammation” that recruits immune cells for fracture repair. Conversely, chronic upregulation fuels pathological tissue destruction or aberrant new bone formation, as seen in Ankylosing Spondylitis (AS) and spinal cord injury sequelae (Chen et al., 2023b) (Table 4).

**TABLE 4:** Summary of key aspects of the Piezo1-mediated inflammatory-stress axis.

Aspect	Physiological/Acute role	Pathological/Chronic implication	Key pathways
Ca^2+^ Overload, ER Stress and mtDNA Release	Transient signaling activates the adaptive UPR to restore homeostasis	Sustained overload triggers CHOP and cGAS-STING axes, driving inflammation and apoptosis	SERCA Overload, PERK/IRE1/ATF6, PERK-CHOP, cGAS-STING
NF-κB Pathway	Short-term activation induces repair factors and immune cell recruitment	Chronic upregulation of IL-1β/MMP13 leads to synovitis and fibrosis	Ca^2+^-IKK-IκB Degradation, ROS-mediated activation
NLRP3 Inflammasome	Acute clearance of cellular debris and damage	Storm-like release of IL-1β exacerbates bone resorption and tissue degeneration	NF-κB/NLRP3 priming, ROS accumulation
Cell death modes	Controlled apoptosis maintains tissue turnover and homeostasis	Ferroptosis/Pyroptosis amplifies inflammation and promotes osteonecrosis	Caspase-3; GPX4 Depletion; GSDMD Pore formation
Temporal dynamics	Acute protection: immune recruitment facilitates fracture healing	Chronic detriment: feedback loops drive progressive tissue degeneration	Mechano-inflammo-metabolic cycle

### 3.5 The transcriptional regulatory axis

**FIGURE 3:**
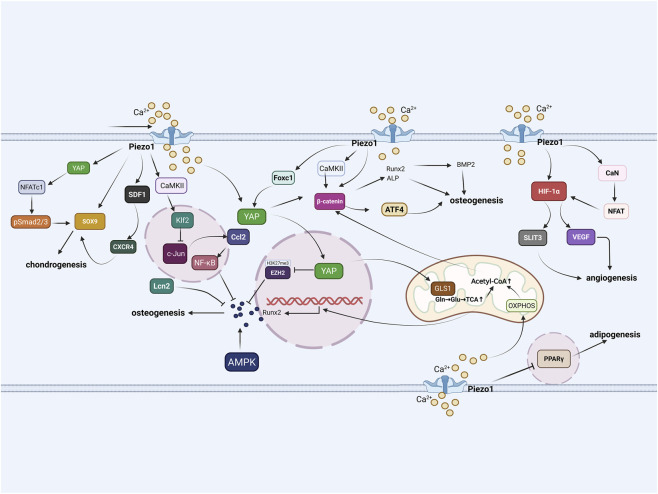
The Piezo1-Mediated transcriptional regulatory network orchestrating cell fate.

Positioned at the core of the mechanotransduction apparatus, Piezo1 functions as a master orchestrator within the transcriptional regulatory network of bone tissue. It translates physical cues into gene expression programs by modulating specific transcription factors through multi-axis integration. As the master regulator of osteogenic differentiation, Runx2 upregulates genes such as *Osterix* and *BMP2* to promote mineralization. Its expression and activity are finely tuned by Piezo1 through diverse mechanisms. In bone defect repair, Piezo1 activation significantly promotes YAP nuclear nucleation and lysine histone acetylation, enhancing YAP’s nuclear function and upregulating Runx2 to accelerate osteogenesis (Liu J. et al., 2025). Similarly, during orthodontic tooth movement, local tension elevates Piezo1, which increases YAP and β-catenin levels, driving Runx2 and ALP upregulation in periodontal ligament stem cells (PDLSCs) (Du Y. et al., 2025). Mechanistically, Piezo1-mediated YAP nuclear entry represses EZH2, thereby reducing the suppressive histone mark H3K27me3 on the Runx2 promoter (Gao et al., 2025). Piezo1 can also upregulate Runx2 via the activation and overexpression of FoxC1, which binds to downstream YAP signaling (Li Z. et al., 2024). Piezo1 activation triggers Ca^2+^ influx and YAP translocation, which promotes GLS1-mediated glutaminolysis. This metabolic shift enhances intracellular Acetyl-CoA production, facilitating Histone H3 acetylation and subsequent *Runx2* transcription (Zhong et al., 2023). Runx2 activity is further modulated by a dynamic equilibrium: it is enhanced by AMPK phosphorylation (Metabolic Axis) but potentially inhibited by NF-κB (Inflammatory Axis) (Wu et al., 2023). Additionally, Piezo1 suppresses adipogenesis and promotes osteogenesis via the CaMKII-Klf2 pathway, which inhibits c-Jun activation and reduces Ccl2/NF-κB -mediated Lipocalin-2 (*Lcn2*) expression (Wang B. et al., 2025). β-catenin serves as a critical node for Piezo1-mediated osteogenesis. In simulated microgravity models, Piezo1 activation rescues bone loss by stimulating β-catenin and its target gene *ATF4*, promoting BMSC proliferation and differentiation (Hu et al., 2023). During fracture healing, Piezo1 enhances YAP expression and nuclear localization in periosteal stem cells. Crucially, YAP interacts directly with β-catenin in the nucleus to form a transcriptional YAP/β-catenin complex, which upregulates osteogenic, chondrogenic, and angiogenic factors (Liu et al., 2022). Mechanistically, Piezo1-induced intracellular Ca^2+^ boosts mitochondrial Oxidative Phosphorylation (OxPhos), significantly elevating cytoplasmic Acetyl-CoA levels. This metabolite is essential for the acetylation and stabilization of β-catenin protein, thereby sustaining osteogenesis (Zhang Q. et al., 2024). Compressive stress activates the Piezo1/CaMKII/β-catenin axis, promoting the mineralization of adipose-derived stem cells (ADSCs), highlighting the role of Ca^2+^-dependent phosphorylation at the β-catenin Ser552 site (Tan et al., 2025). In cartilage, Piezo1 is pivotal for maintaining the chondrocyte phenotype via SOX9. In mandibular condylar chondrocytes, Piezo1 activation under cyclic tensile strain is essential for maintaining SOX9 and Type II collagen (COL2A1) protein levels; Piezo1 knockdown leads to their significant downregulation (Zhang Z. et al., 2022). In Osteoarthritis (OA) models, mechanical loading promotes endogenous stem cell migration and chondrogenic differentiation via the Piezo1-mediated SDF-1/CXCR4 axis, accompanied by SOX9 upregulation (Sun et al., 2023). In meniscus regeneration, biomechanical stiffness upregulates Piezo1, which triggers the synergistic activation of Calcineurin and NFATc1. This subsequently activates the YAP-pSmad2/3-SOX9 axis, driving fibrochondrogenic differentiation of MSCs (Yan et al., 2023). However, regulation is context-specific; under inflammatory conditions caused by aberrant loading, Piezo1 activation may be linked to chondrocyte pyroptosis, suggesting a pathological deviation of this pathway (Zhang Y. et al., 2025). Piezo1 acts as a potent repressor of PPARγ, a key regulator of adipogenesis, thereby governing the lineage commitment of BMSCs. Activation of Piezo1 suppresses PPARγ expression, inhibiting lipid accumulation. This is evident in thyroid eye disease research, where Piezo1 activation reduces adipogenic transcription factors (Galgoczi et al., 2025). The age-related decline in Piezo1 activity correlates with increased PPARγ expression, providing a mechanistic explanation for the accumulation of marrow adipose tissue (MAT) and reduced osteogenesis observed in aging skeletons (Byun et al., 2024). As a critical hypoxic response factor, HIF-1α is upregulated by Piezo1-mediated mechanical signals to coordinate tissue repair. In alveolar bone homeostasis, occlusal force activates the Piezo1/Ca^2+^/HIF-1α/SLIT3 axis in periodontal ligament cells, promoting the formation of Type H vessels and the function of osteogenic progenitors (Chen Y. et al., 2023). In steroid-induced osteonecrosis of the femoral head (ONFH), mechanical vibration therapy activates Piezo1 to upregulate the HIF-1α/VEGF axis, improving intraosseous microcirculation and osteogenic differentiation (Tian et al., 2023). Piezo1-mediated HIF-1α signaling also modulates the bone marrow microenvironment, such as promoting VEGFA release from macrophages via the CaN/NFAT/HIF-1α pathway (Zhang X. et al., 2022; Wang et al., 2025e) (Figure 3) (Table 5).

**TABLE 5:** Summary of key aspects of the Piezo1-mediated transcriptional regulatory network.

Target transcription factor	Physiological role/Outcome	Pathological implication	Key regulatory mechanisms
Runx2 Regulation	Promotes osteogenic differentiation; upregulates *Osterix/BMP2/ALP*; accelerates mineralization; prevents adipogenesis	Deficiency leads to impaired bone formation and exacerbated osteoporosis	YAP nucleation and Lysine acetylation; EZH2 inhibition (↓H3K27me3); FoxC1-YAP interaction; GLS1-mediated Glutaminolysis/H3 acetylation; Inhibition of Ccl2-Lcn2 axis
β-catenin Regulation	Stabilizes nuclear translocation; forms YAP/NFAT complexes; promotes proliferation, fracture healing, and microcirculation	Dysregulation exacerbates bone loss and delays fracture healing	AKT/GSK3β-mediated stabilization; YAP/β-catenin transcriptional complex; CaMKII-dependent phosphorylation (Ser552); Acetyl-CoA-mediated stabilization
SOX9 Regulation	Maintains chondrogenic phenotype; drives fibrochondrogenic differentiation; facilitates meniscus repair	Upregulation inhibits osteogenesis; linked to cartilage degeneration and pyroptosis	Cyclic tension maintains SOX9/COL2A1; SDF-1/CXCR4 axis activation; CaN/NFAT/YAP-pSmad2/3-SOX9 axis; Aberrant loading activates pyroptosis
PPARγ Regulation	Inhibits adipogenic differentiation; “Locks” lineage commitment to osteogenesis; maintains skeletal homeostasis	Upregulation drives bone marrow adipose tissue (MAT) accumulation and age-related bone loss	Inverse correlation: Piezo1 activation suppresses PPARγ and lipid accumulation; Age-related Piezo1 decline increases PPARγ expression
HIF-1α Regulation	Promotes hypoxic adaptation, Type H vessel formation, and osteoprogenitor function; improves microcirculation	Hyperactivation may contribute to bone resorption and degeneration	Piezo1/Ca^2+^/HIF-1α axis (Type H vessels); Vibration-induced HIF-1α/VEGF axis; CaN/NFAT/HIF-1α-mediated VEGFA release

### 3.6 Network-level crosstalk among Piezo1-dependent axes

Piezo1-dependent mechanotransduction is best understood as a networked response rather than a set of isolated cascades. Calcium entry represents the first convergence node. A single burst of Piezo1-mediated Ca^2+^ influx can simultaneously activate calcineurin/NFAT signaling, CaMKII-dependent phosphorylation, RhoA/ROCK-mediated cytoskeletal remodeling, and mitochondrial calcium uptake. These calcium-dependent events then diverge into different but interacting outputs. Calcineurin and CaMKII regulate transcriptional programs, whereas RhoA/ROCK and actin polymerization promote YAP/TAZ nuclear localization. Mitochondrial calcium uptake provides a second convergence node by increasing tricarboxylic acid cycle activity and ATP generation, thereby supporting cytoskeletal remodeling and matrix synthesis. However, excessive or sustained mitochondrial calcium accumulation may elevate ROS production, lower the threshold for NF-κB and NLRP3 activation, and shift the cellular response from adaptive remodeling to inflammatory injury. YAP/TAZ serves as another central integrator. Its nuclear localization is influenced not only by cytoskeletal tension but also by calcium-dependent signaling and metabolic status. Once in the nucleus, YAP/TAZ interacts with Runx2, β-catenin, TEADs, and other transcriptional regulators to coordinate osteogenesis, chondrogenesis, angiogenesis, and matrix remodeling. Conversely, inflammatory signaling can antagonize osteogenic transcriptional programs by activating NF-κB and stress-response pathways. Thus, Piezo1-mediated signaling should be interpreted as a dynamic balance among adaptive calcium signaling, mechanical reinforcement of the cytoskeleton, metabolic compensation, and stress-induced inflammatory amplification. This network-level view explains how similar Piezo1 activation can support bone formation under physiological loading but promote cartilage degeneration, apoptosis, ferroptosis, or pyroptosis under excessive or chronic mechanical stress.

## 4 Piezo1 in orthopedic diseases

### 4.1 Osteoporosis: the decline of mechanosensation

Osteoporosis is a systemic skeletal disorder characterized by reduced bone mass and microarchitectural deterioration, fundamentally driven by a disruption in the dynamic equilibrium between bone formation and resorption. With advancing age (senile osteoporosis) or reduced mechanical loading (disuse osteoporosis, e.g., prolonged bed rest, immobilization, or microgravity), the sensitivity of bone tissue to mechanical stimuli declines significantly (Hu et al., 2023; Beyersdorf et al., 2025). Emerging evidence implicates the functional decline or downregulation of the mechanosensitive ion channel Piezo1 as a critical molecular event underlying this loss of mechanosensitivity and the subsequent uncoupling of bone remodeling. When Piezo1 fails to effectively sense mechanical loads, downstream signaling networks undergo maladaptive alterations, ultimately precipitating bone loss (Sun et al., 2019; Beyersdorf et al., 2025). Buried deep within the bone matrix, osteocytes form the lacunar-canalicular network (LCN) via their extensive dendritic processes and serve as the primary mechanosensors of the skeleton. Under physiological conditions, load-induced interstitial fluid flow generates shear stress that activates Piezo1 channels on the osteocyte membrane, triggering intracellular Ca^2+^ influx and initiating downstream cascades. However, in the pathological progression of osteoporosis, osteocytic Piezo1 function is often compromised (Wu et al., 2025b). Studies indicate that age-related downregulation of Piezo1 directly impairs the mechanoreactivity of bone tissue, driving the pathogenesis of senile osteoporosis (Liu C. et al., 2025). When Piezo1 activation is blunted, both the ionic cascade axis (Ca^2+^ signaling) and the cytoskeletal-nuclear axis (YAP/TAZ signaling) fail to initiate effectively. A critical consequence of this transduction failure is the dysregulation of the OPG/RANKL ratio:Physiological State: Mechanically activated Piezo1 promotes Osteoprotegerin (OPG) secretion and inhibits RANKL expression. OPG acts as a decoy receptor, preventing RANKL from binding to RANK on osteoclast precursors, thereby suppressing osteoclast differentiation (Han et al., 2018). Osteoporotic State: Piezo1 dysfunction lowers the OPG/RANKL ratio, releasing the “brake” on osteoclasts and promoting resorption. Furthermore, Sclerostin (SOST), a potent Wnt signaling inhibitor secreted by osteocytes, binds to LRP5/6 receptors on osteoblasts to block Wnt/β-catenin signaling. While mechanical loading typically downregulates SOST via Piezo1-dependent mechanisms, mechanical insufficiency or Piezo1 dysfunction leads to elevated SOST levels, strongly suppressing osteoblast function (Wu et al., 2021). Thus, the sensory failure of osteocytic Piezo1 directly drives increased resorption and diminished formation. Beyond osteocytes, Piezo1 plays a vital role in BMSCs and osteoblasts by sensing matrix stiffness and tensile forces to drive osteogenic differentiation and maintain matrix synthesis (Hu et al., 2023). In osteoporotic states, due to reduced loading or cellular senescence, Piezo1 signaling activity diminishes. Lacking Piezo1-mediated Ca^2+^ and YAP/TAZ signals, the expression of key osteogenic transcription factors like Runx2 declines (Hao et al., 2024). Conversely, the activity of the adipogenic transcription factor PPARγ may relatively increase, skewing BMSC lineage commitment toward adipocytes rather than osteoblasts—a phenomenon known as the “adipo-osteogenic switch”—thereby depleting the pool of osteoblast progenitors (Huang et al., 2025). Recent insights also reveal a novel role for Piezo1 in integrating bone metabolism with other systems. Macrophage Piezo1 in the bone marrow senses mechanical changes and promotes VEGFA release via the CaN/NFAT/HIF-1α pathway, facilitating Type H vessel formation coupled with osteogenesis (Zhang X. et al., 2022). Piezo1 dysfunction compromises this vascular support, further impacting bone mass (Wang et al., 2024; Guan et al., 2024). Intriguingly, Piezo1 in intestinal epithelial cells also regulates bone metabolism. Deletion of intestinal Piezo1 lowers systemic serotonin (5-HT) levels, relieving the inhibition of osteoblast proliferation by gut-derived serotonin (Sugisawa et al., 2020). This suggests Piezo1 may regulate skeletal homeostasis remotely, implying that osteoporosis involves the dysregulation of a broader, systemic mechanotransduction network. In summary, Piezo1 acts as a “central failure node” in osteoporosis. Its functional decline across osteocytes, the osteogenic lineage, and systemic regulators precipitates a comprehensive failure of mechanotransduction, triggering downstream signaling dysregulation that ultimately shatters the balance of bone remodeling.

### 4.2 Fracture healing: mechanical force-guided tissue regeneration

Fracture healing is a complex process heavily reliant on mechano-biological coupling, encompassing inflammation, chondrogenesis, endochondral ossification, and bone remodeling. Accumulating evidence identifies Piezo1 as a pivotal mechanosensor within the fracture callus, orchestrating the functions of multiple cell lineages to dictate the kinetics and quality of repair. Piezo1 is highly enriched in Periosteal Stem Cells (PSCs) and their downstream osteo/chondrogenic lineages. By promoting YAP expression and nuclear translocation as well as β-catenin activation, Piezo1 drives the chondro-osteogenic transdifferentiation of PSCs, thereby accelerating callus formation and the transition from cartilage to bone. Downregulation of Piezo1 impedes healing, whereas activation with the specific agonist Yoda1 significantly improves callus mineralization and mechanical properties in delayed healing models, highlighting the conserved and requisite role of Piezo1 in load-dependent repair (Liu et al., 2022). Regarding endochondral ossification, recent findings indicate that Piezo1 deficiency impairs the transdifferentiation of chondrocytes into osteoblasts, delaying ossification. Mechanistically, Piezo1 loss disrupts mitochondrial bioenergetics, characterized by reduced membrane potential, diminished ATP production, lowered oxygen consumption rate (OCR), and elevated mitochondrial ROS. Single-cell transcriptomics (scRNA-seq) revealed an upregulation of LARS2 in these dysfunctioning chondrocytes; inhibiting LARS2 restored mitochondrial homeostasis and reversed the downregulation of osteogenic markers caused by Piezo1 deficiency. This suggests that Piezo1 facilitates healing by maintaining mitochondrial function and powering the metabolic demands of the cartilage-to-bone transition (Zhang T. et al., 2025). Piezo1 in Endothelial Cells (ECs) is indispensable for the vascularization phase of healing. Endothelial-specific deletion of Piezo1 inhibits angiogenesis at the fracture site and reduces calcium-dependent Calpain activity. This impairment compromises downstream PI3K-AKT and Notch signaling, indirectly suppressing osteoblast maturation and bone formation, ultimately leading to non-union or poor healing (Chen et al., 2021). These findings underscore the critical position of Piezo1 in mediating osteogenesis–angiogenesis coupling (specifically Type H vessel formation). Several studies provide direct evidence for targeting Piezo1 via exogenous mechanical stimulation to enhance healing. High-intensity ultrasound (surpassing traditional LIPUS) significantly promotes fracture healing, an effect attenuated by the Piezo1 inhibitor GsMTx4. *In vitro* knockdown of Piezo1 in osteocytes/osteoblasts also diminishes ultrasound-induced osteogenic gene expression, confirming Piezo1 as a primary sensor for therapeutic ultrasound (Guo et al., 2024; Inoue et al., 2023). Engineering strategies employing “micromotion”, such as NIPAM/Nb_2_C thermo-responsive hydrogels, generate precise frequency-dependent micro-deformations. Optimal micromotion frequencies activate Piezo1 to promote BMSC osteogenic differentiation and accelerate bone regeneration *in vivo* (Yang et al., 2024). This suggests a promising avenue for combining material design with Piezo1-targeted mechanotherapy. In the realm of translational medicine, scRNA-seq and drug screening have identified natural small molecules, such as Asperosaponin VI, which can bind to Piezo1. This binding enhances the osteogenic potential of LEPR^+^ BMSCs and promotes local angiogenesis and osteo-vascular coupling, effectively improving healing in osteoporotic fracture models. This provides preliminary molecular and cellular evidence for developing Piezo1-targeted pharmacotherapies (Dong et al., 2025).

### 4.3 The “double-edged sword” in osteoarthritis (OA): shifting from homeostasis to pathological destruction

Osteoarthritis (OA) is defined as a “whole-joint” disease, characterized primarily by articular cartilage degeneration but inextricably linked with osteophyte formation, synovial inflammation, meniscus degeneration, subchondral bone remodeling, and pathological alterations in the infrapatellar fat pad (Hunter et al., 2020). When articular cartilage and synovium are exposed to physiological mechanical stimuli, Piezo1 acts as a sentinel for cellular homeostasis, metabolic activity, and local regenerative capacity. Moderate loading activates Piezo1 to maintain intracellular Ca^2+^ homeostasis and promote matrix synthesis. Crucially, it sustains the renewal of superficial Procr^+^ mechanosensitive progenitors, serving as a vital node for healthy mechano-regenerative coupling (Zhu et al., 2025). In rat models, mild mechanical loading elicits a protective response via Piezo1, enhancing the expression of anti-inflammatory factors (IL-10, TGF-β) while suppressing pro-inflammatory signals to maintain the joint’s inflammation-repair equilibrium. This protective mechanism involves fine-tuned regulation: moderate Ca^2+^ influx activates the cAMP pathway, boosting cell survival signals and engaging negative feedback loops to dampen excessive inflammation (Zhou et al., 2026). Furthermore, endogenous factors like Urocortin-1 (Ucn1) reinforce this physiological protection. Ucn1 binds to Piezo1 via the CRF-R1 receptor, preventing the non-selective over-opening of the channel and blocking basal Ca^2+^ accumulation, thereby shielding chondrocytes from stress and halting OA progression (Jones et al., 2022; Lawrence et al., 2017). Dynamic interactions with the extracellular matrix (ECM) further ensure adaptive responses, promoting ECM synthesis and tissue elasticity (Zhao T. et al., 2025). Under these conditions, Piezo1 functions effectively as a “Homeostatic Regulator”, preventing microdamage accumulation and supporting joint function. However, during the onset and progression of OA, aberrant mechanical stress patterns flip the switch, converting Piezo1 from a “maintainer of homeostasis” to a “promoter of destruction”. Overload or shear stress triggers Piezo1 hyperactivation, causing a “calcium flood” that induces mitochondrial damage and mtDNA release. This activates the cGAS–STING pathway, driving the spread of synovial inflammation and cartilage degradation (Sun L. et al., 2025). When mechanical stress exceeds physiological thresholds (e.g., chronic overload or trauma) and intersects with an inflammatory milieu, Piezo1 rapidly pivots to a destructive role, becoming a core driver of OA pathology. Clinical and experimental evidence confirms that Piezo1 upregulation in OA cartilage correlates positively with disease severity. In human OA samples, mRNA and protein levels are significantly elevated, a result of transcriptional reprogramming induced by inflammatory factors like IL-1β (Han et al., 2025; Lee et al., 2021). In load-induced OA models, Piezo1-mediated aberrant Ca^2+^ influx triggers the PI3K/AKT/mTORC1 and NF-κB pathways. This shifts the cytokine profile—upregulating pro-inflammatory IL-1β, IL-6, and TNF-α while downregulating anti-inflammatory IL-10 and TGF-β—ultimately accelerating matrix degradation (Zhou et al., 2026; Han et al., 2025; Wu et al., 2025c). Metabolically, Piezo1 activation enhances HK2-dependent glycolysis and initiates a MIF-Th17 immune program, amplifying the inflammatory phenotype and expanding joint destruction (Zhou et al., 2025b). The pathological reach of Piezo1 extends to nociception and structural remodeling. Within the synovium-infrapatellar fat pad unit, Piezo1 synergistically promotes angiogenesis and macrophage infiltration, sensitizing nerve endings and exacerbating OA-related pain (Emmi et al., 2022). Chondrocyte-specific knockout of Piezo1 significantly attenuates degeneration, synovitis, and pain behavior, confirming its pivotal status in pathological destruction (Ely et al., 2025). The Mechanism of Transition The transition from protection to destruction hinges on threshold effects and a complex mechano-inflammatory feedback loop. When loading exceeds the threshold, Piezo1 signaling shifts from transient Ca^2+^ pulses to sustained influx. Inflammatory factors sensitize Piezo1 expression and remodel the cytoskeleton, further amplifying mechanosensitivity (Lee et al., 2021). The imbalance of negative regulators is a key tipping point. For instance, the G-protein coupled estrogen receptor (GPER) normally inhibits Piezo1 activity via the YAP/ARHGAP29/RhoA/LIMK/cofilin pathway to stabilize actin and maintain homeostasis; its downregulation in OA unleashes Piezo1 hyperactivity (Sun et al., 2021). Piezo1 and Piezo2 exhibit synergistic redundancy in pathology; while single knockout offers partial relief, double knockout provides superior cartilage protection (Ely et al., 2025). The “double-edged sword” effect of Piezo1 in OA highlights the profound complexity of mechanosensation in cell fate regulation. It provides a novel paradigm: a shift from homeostatic maintenance to pathogenic driving forces mediated by threshold-sensitive networks, inflammatory feedback, and multicellular crosstalk.

### 4.4 Intervertebral disc degeneration (IVDD)

Intervertebral Disc Degeneration (IVDD) is the leading cause of low back pain, affecting over 600 million people globally. As the critical functional unit of the spine, the intervertebral disc (IVD) endures complex mechanical loads, and aberrant mechanical stress is widely recognized as a primary pathogenic factor. Recent breakthroughs have identified Piezo1 as a central player in IVDD pathogenesis, where its dysregulation constitutes a “pathological bridge” linking mechanical injury to degenerative progression (Li et al., 2025). Piezo1 transduces abnormal axial compression, impact, or shear forces into sustained intracellular Ca^2+^ dysregulation. This, in turn, activates a spectrum of pathological axes—including the NLRP3 inflammasome, mitochondrial dysfunction, ER stress, autophagy inhibition, mTOR hyperactivation, and epigenetic reprogramming—forming a self-amplifying degenerative cascade (Chen et al., 2026). Piezo1 is significantly upregulated in degenerated disc tissues. In rat models of lumbar instability, Piezo1 expression in the Nucleus Pulposus (NP) increases in parallel with the degree of degeneration. Similarly, in human patients, Piezo1 levels in NP cells correlate positively with degeneration severity (Sun H. et al., 2025; Shi et al., 2022). Transcriptomic analysis has identified distinct molecular subtypes in IVDD patients; notably, the C1 subtype chondrocytes drive collagen production primarily via the activation of mechanosensors TRPV4 and Piezo1 (Jin et al., 2025). Crucially, *in vivo* knockdown of Piezo1 or treatment with the antagonist GsMTx4 significantly mitigates IVDD induced by instability or aging, reducing ECM degradation and NP cell apoptosis (Li et al., 2025). This confirms that Piezo1 is not merely a passive sensor but an active driver of IVDD. Piezo1 drives IVDD through multiple distinct but interconnected pathways: Aberrant stress activates the Piezo1/Calpain2/BAX/Caspase-3 pathway, inducing apoptosis in Annulus Fibrosus Cells (AFCs) (Liu C. et al., 2023). Mechanical stretch promotes Piezo1 and NLRP3 expression in a time-dependent manner. Piezo1 facilitates NLRP3 inflammasome assembly via the Ca^2+^/NF-κB pathway, revealing a novel mechanism for inflammation-driven progression (Sun et al., 2020). Even a single impact injury can trigger degeneration via Piezo1-mediated upregulation of NLRP3, catabolic enzymes (MMP-9, MMP-13), and pro-inflammatory cytokines (IL-1β) (Sun et al., 2022). In Cartilage Endplate (CEP) cells, excessive compression induces degeneration via the Piezo1/NAT10/mTOR axis. Mechanistically, Piezo1 enhances the transcriptional activity of the NAT10 promoter via P65, establishing a “mechanosensing-transcription loop”. Upregulated NAT10 stabilizes mTOR via ac4C modification, thereby inhibiting autophagy and accelerating apoptosis and ECM degradation (Sun H. et al., 2025). Piezo1-mediated Ca^2+^ overload causes mitochondrial membrane potential collapse, ROS bursts, and increased iron influx, inducing ferroptosis in NP cells independently of the classical transferrin receptor (TFRC) (Xiang et al., 2024). Selenium supplementation counters this process. Selenium upregulates Selenoprotein K (SelK) to alleviate ER stress and lower cytosolic Ca^2+^. Via the Se-GPX4 and Se-SelK axes, it restores GPX4 function and stabilizes the ECM, offering a potential therapeutic strategy (Jia C. et al., 2024). ECM stiffness acts as a critical mechanical cue regulating mitochondrial dynamics via Piezo1. Stiff matrices activate the ERK1/2 pathway, increasing Drp1 phosphorylation at Ser616. This promotes excessive mitochondrial fission and apoptosis in NP cells. Inhibition of the Piezo1-ERK1/2 axis effectively reduces stiffness-induced ROS and apoptosis (Ke et al., 2023). This highlights the pathological significance of progressive matrix stiffening in IVDD, where Piezo1 converts physical stiffness into mitochondrial dysfunction. In summary, Piezo1 has evolved from a simple “mechanosensor” to a “pathological central hub” in IVDD. Its sustained activation efficiently converts aberrant mechanical loads into multi-pathway synergistic damage—calcium signaling, inflammasome activation, mitochondrial dysfunction, autophagy inhibition, and ferroptosis. This represents the critical node where IVDD transitions from reversible mechanical sensing to the irreversible malignant transformation of cell fate.

## 5 Therapeutic strategies targeting Piezo1 and future perspectives

### 5.1 Piezo1 agonists: mimicking exercise to rebuild skeletal health

Whether stemming from microgravity (spaceflight), prolonged bed rest, paralysis due to spinal cord injury, or simply the decline in physical capacity associated with aging, the absence of mechanical loading precipitates rapid bone loss (Hao et al., 2024). The etiology of this “disuse osteoporosis” lies in the functional quiescence of the mechanotransduction network within bone tissue. The advent of Piezo1 agonists allows us to bypass the dependency on physical force and directly activate downstream osteogenic signaling networks at the molecular level. This represents a revolutionary leap in the therapeutic philosophy of osteoporosis management (Beyersdorf et al., 2025). The small molecule Yoda1 stands as the most extensively characterized specific agonist of Piezo1. It functions by lowering the mechanical threshold for channel opening or directly stabilizing the open conformation, effectively simulating the effects of mechanical stimulation (Botello-Smith et al., 2019). In animal models of osteoporosis, Yoda1 administration has been proven to effectively activate Piezo1 signaling in osteocytes and osteoblasts, promoting Ca^2+^ influx to “wake up” bone cells. This action prevents bone loss and significantly enhances bone mass and mechanical strength (Meslier et al., 2025). Long-term use of glucocorticoids (e.g., dexamethasone) is a leading cause of secondary osteoporosis. Mechanistically, dexamethasone upregulates the transcriptional repressor Hes1, which directly silences *Piezo1* transcription, rendering the skeleton “desensitized” to mechanical stimuli. Systemic administration of Yoda1 acts as a dual-action rescuer: it not only activates residual Piezo1 channels but also restores *Piezo1* expression levels via a positive feedback mechanism. Crucially, Yoda1 reactivates the Akt and CaMKII signaling axes that were suppressed by dexamethasone. This molecular reactivation restores the integrity of the damaged lacunar-canalicular network (LCN), downregulates Sclerostin, and corrects the RANKL/OPG imbalance. Thus, Yoda1 not only promotes osteogenesis but also curbs the hyperactive bone resorption typical of GIOP (Ochiai et al., 2024). Beyond its potential in treating osteoporosis, Yoda1 demonstrates broad therapeutic applicability in other scenarios characterized by mechanical deficits or high regenerative demands. Systemic administration of Yoda1 has been shown to effectively mimic mechanical stimuli, significantly alleviating bone loss induced by unloading in microgravity environments (Hu et al., 2023). Furthermore, in the context of fracture healing, Yoda1 facilitates the repair process by promoting the recruitment and osteogenic differentiation of periosteal stem cells, thereby accelerating callus formation and osseous consolidation (Liu et al., 2022). Yoda1 has been widely used as a Piezo1 agonist and has greatly facilitated mechanistic studies, but it does not fully recapitulate the spatial directionality, temporal dynamics, and multimodal nature of physiological mechanical loading. In addition, its solubility, stability, and context-dependent pharmacological behavior require careful experimental control. Building upon the scaffold of Yoda1, rational drug design has yielded next-generation agonists such as MCB-22-174, which appears to induce more sustained activation of the CaMKII/ERK1/2 axis. Compared to its predecessor, MCB-22-174 demonstrates superior efficacy in improving trabecular microarchitecture, and its optimized physicochemical properties render it more suitable for oral or long-acting injectable formulations, thereby opening avenues for the chronic treatment of disuse osteoporosis (Hao et al., 2024). Concurrently, fundamental research into Piezo1 gating has expanded the agonist landscape with the discovery of the Jedi series. Unlike Yoda1, which targets the “Beam” domain, Jedi1/2 bind to the massive extracellular “Blade” structure. Although currently utilized primarily as research tools—with Jedi2 exhibiting higher selectivity for Piezo1 over Piezo2—their distinct binding sites theoretically offer potential for synergistic effects with Yoda1 and enrich the agonist pharmacophore library, paving the way for future “cocktail therapies” (Wang et al., 2018). Collectively, the emergence of these Piezo1 agonists offers the promising concept of “Exercise in a pill”. By circumventing the physical medium of force to simulate mechanobiological effects directly at the molecular level, these agents hold the potential to maintain skeletal health in populations incapable of effective physical exercise, holding profound strategic significance for the elderly, bedridden rehabilitation patients, spinal cord injury victims, and astronauts on long-duration space missions.

### 5.2 Piezo1 inhibitors: intercepting pathological signaling for tissue preservation

Under certain pathological conditions, excessive mechanical stimulation or sustained hyperactivation of Piezo1 leads to cellular injury and tissue degeneration, necessitating inhibitory strategies. The Biological Prototype for Inhibition GsMTx4, a peptide derived from tarantula venom, stands as the only Piezo1 inhibitor repeatedly validated for efficacy across various orthopedic disease models and is regarded as the agent closest to clinical translation. Rather than binding directly to the channel pore, it acts as a gating modifier by inserting into the lipid bilayer and altering membrane tension, thereby inhibiting the mechanogating activity of Piezo1 (Bae et al., 2011). In the context of OA, chondrocytes undergo aberrant mechanical compression, leading to Piezo1 hyperactivation, intracellular Ca^2+^ overload, and massive inflammatory cytokine release. Intra-articular injection of GsMTx4 effectively dampens these pathological mechanical responses, reducing inflammatory mediators and retarding cartilage degeneration, demonstrating significant chondroprotective effects (Ren et al., 2023). Furthermore, inhibiting Piezo1 activity upregulates GPX4 expression, attenuating the ferroptosis phenotype to reduce OA severity (Wang et al., 2022). Under inflammatory conditions, Piezo1 drives excessive mitochondrial fission via the Ca^2+^/CaMKII/Drp1 axis. GsMTx4 significantly restores mitochondrial morphology and inhibits senescence and apoptosis; intradiscal injection has been shown to delay Cartilage Endplate (CEP) degeneration in rat models (Lin Z. et al., 2025). Following tendon injury, Piezo1 activation promotes the chondrogenic differentiation of Tendon-Derived Stem Cells (TDSCs). Application of a GsMTx4-loaded GelMA hydrogel significantly promotes tendon regeneration and inhibits Heterotopic Ossification (HO) by blocking the Apelin signaling pathway (Lei et al., 2026). As a selective Mechanosensitive Ion Channel (MSIC) inhibitor, GsMTx4 reversibly blocks mechanically induced electrical activity. Intra-articular application significantly suppresses mechanosensitized nociceptor activity and attenuates pain circuits in the spinal dorsal horn, elevating the mechanical pain threshold in pathological states (He et al., 2017). Mechanical force is a key regulator of scar formation. Cyclic Mechanical Stretching (CMS) increases Piezo1-mediated calcium influx in dermal fibroblasts, while intradermal injection of GsMTx4 significantly inhibits hypertrophic scar formation (He et al., 2021). Pharmacologically, Yoda1 was the first widely used small-molecule agonist of Piezo1 and has become an important tool for investigating Piezo1-dependent signaling pathways. As a structural analogue of Yoda1, Dooku1 antagonizes Yoda1-induced Piezo1 activation and is therefore useful for distinguishing Yoda1-dependent pharmacological effects from other Piezo1-related responses. However, Dooku1 should not be regarded as a general Piezo1 blocker, because its inhibitory activity is mainly directed against Yoda1-induced activation rather than constitutive or mechanically evoked Piezo1 activity. Similarly, GsMTx4 should not be interpreted as a strictly Piezo1-specific antagonist. Instead, it primarily acts as a gating modifier of mechanosensitive channels by altering the local lipid bilayer environment and may therefore affect multiple mechanically activated cation channels. Accordingly, pharmacological activation or inhibition of Piezo1 should be interpreted with caution and, whenever possible, validated using genetic approaches such as PIEZO1 knockdown, conditional knockout, or rescue experiments. While GsMTx4 represents the biological frontier, the identification of Artemisinin—the Nobel Prize-winning antimalarial drug—as a Piezo1 inhibitor offers a promising avenue for drug repurposing. Recent screenings unexpectedly revealed that Artemisinin and its derivatives efficiently inhibit Piezo1-mediated calcium influx. Unlike GsMTx4, which requires local injection, Artemisinin is a small molecule with excellent oral bioavailability and a long-standing safety record in humans (Ma et al., 2020). In DMM-induced OA models, Artemisinin exhibits protective effects comparable to chondrocyte-specific Piezo1 knockout. Mechanistically, it inhibits the PI3K/AKT signaling pathway, thereby attenuating cell hypertrophy and catabolism triggered by Piezo1 hyperactivation, reducing RUNX2 and MMP13 expression, and preserving Type II collagen levels (Gan et al., 2023). Many *in vitro* studies are performed on rigid plastic or glass substrates, whose stiffness is far from that of native skeletal tissues. Such artificial mechanical environments may influence basal cytoskeletal tension, YAP/TAZ localization, Piezo1 sensitivity, and downstream signaling thresholds. Future studies should incorporate tunable hydrogels, three-dimensional matrix systems, organoids, explant cultures, and well-calibrated mechanical loading platforms to better mimic the native bone and cartilage microenvironment.

### 5.3 Molecular targets of physical therapy: the pivotal role of Piezo1 in low-intensity pulsed ultrasound (LIPUS)

**FIGURE 4:**
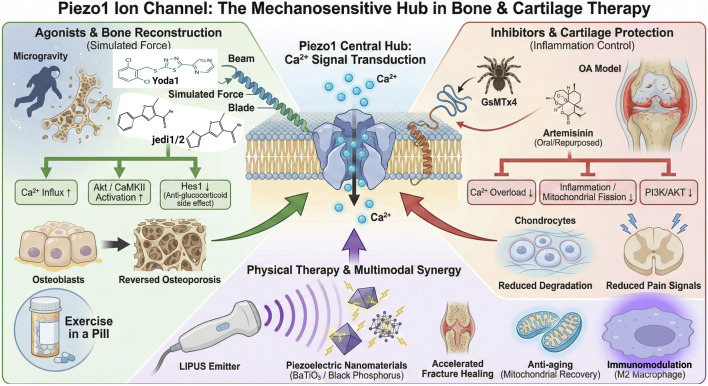
Therapeutic targeting of Piezo1: From molecular agonism to multimodal physical therapy.

Low-Intensity Pulsed Ultrasound (LIPUS), an FDA-approved non-invasive modality, has been definitively shown to drive bone regeneration by activating the mechanosensitive ion channel Piezo1, establishing it as the most well-characterized upstream molecular target in orthopedic physical therapy. Zhang et al. provided the first real-time recordings of Piezo1-mediated oscillatory Ca^2+^ influx in MC3T3-E1 cells following LIPUS stimulation, which subsequently triggered ERK1/2 phosphorylation and rapid perinuclear F-actin polymerization. These effects were abolished by Piezo1 knockdown or GsMTx4 blockade, confirming Piezo1 as a prerequisite for LIPUS-induced mechanotransduction in osteoblasts (Zhang et al., 2021). In murine osteoporotic fracture models, ultrasound at intensities higher than traditional LIPUS significantly accelerated both endochondral and intramembranous ossification. Crucially, this acceleration was completely negated by GsMTx4, providing the first *in vivo* evidence that Piezo1 is an indispensable pathway for ultrasound-mediated fracture healing (Inoue et al., 2023). The therapeutic scope of Piezo1-targeted LIPUS extends beyond simple osteogenesis. In type 1 diabetic mice, LIPUS simultaneously improved bone microarchitecture and significantly lowered blood glucose levels, with efficacy comparable to insulin. Piezo1-knockout cells lost these responses, suggesting that Piezo1-mediated mechanotransduction co-regulates bone and glucose metabolism (Cao et al., 2026). By implanting a BaTiO_3_/MWCNT/collagen biomimetic periosteum into cranial defects, Jiang et al. demonstrated that remote LIPUS activation of Piezo1 drove macrophage polarization toward the M2 phenotype, significantly enhancing bone regeneration (Jiang et al., 2024). In a supraspinatus tear model, LIPUS-mediated reactivation of Piezo1 in Fibro-Adipogenic Progenitors (FAPs) inhibited adipogenesis via the ERK/KLF4 pathway, reducing fatty infiltration and improving shoulder function (Lin X. et al., 2025). Recent advancements have integrated LIPUS with piezoelectric nanomaterials (e.g., BTO or Black Phosphorus) incorporated into PEEK or decellularized matrices. Under LIPUS stimulation, these materials generate local micro-electric fields and mild thermal stimuli that further amplify Piezo1 activation. This mechano-electrical-thermal tri-modal synergy significantly enhances ossification efficiency in craniofacial defects (Jia W. et al., 2025; Li Y. et al., 2024). Interestingly, the role of Piezo1 is nuanced. On the compression side of orthodontic tooth movement, LIPUS promotes PDLC osteogenic differentiation by *downregulating* Piezo1 expression. This highlights the context-dependent, bidirectional nature of Piezo1 in distinct mechanical microenvironments (tension vs. compression), elucidating the unique efficacy of LIPUS in orthodontic root preservation (Zheng et al., 2024). Most recently, the Kureel/Shetz team demonstrated that low-frequency ultrasound induces massive autophagy and inhibits mTORC1 via Piezo1, reversing 15 distinct cellular senescence phenotypes and significantly extending both lifespan and healthspan in mice. This provides compelling evidence for expanding Piezo1 activation strategies to treat senile osteoporosis (Kureel et al., 2025). In summary, Piezo1 has emerged as the definitive molecular switch for physical therapies. Future strategies employing multi-physics precision regulation—combining ultrasound with piezoelectric materials or specific agonists—hold the promise of becoming the “third mainstream pillar” for managing bone metabolic diseases, complementing surgical and pharmacological interventions (Figure 4).

## 6 Challenges and future directions

### 6.1 Breaking the “static stiffness” dogma: the new frontier of viscoelasticity and stress relaxation

For the past two decades, the dominant paradigm in bone tissue engineering and mechanobiology has been governed by “matrix stiffness”, positing that rigid substrates drive osteogenesis while soft substrates favor adipogenesis or chondrogenesis. However, recent research suggests that this view, based on linear elastic materials, may oversimplify the authentic cellular environment. The bone matrix is not an ideal Hookean spring but a complex viscoelastic material. Its mechanical properties change dynamically over time, exhibiting stress relaxation—the dissipation of stress under constant strain. Piezo1 acts as the core sensor for these time-dependent signals. On soft yet fast-relaxing substrates, cellular traction forces generate local, transient peaks in membrane tension, momentarily activating Piezo1. This induces moderate Ca^2+^ oscillations and YAP nuclear translocation, thereby driving cell spreading and osteogenic differentiation (Oliva et al., 2025). This discovery fundamentally overturns the “static stiffness matching” dogma of biomaterial design, propelling the field toward an era of “dynamic mechanical programming”. Cytoskeletal contractile forces directly oppose matrix stiffness. If the matrix is too soft, the molecular clutch cannot build sufficient tension to open Piezo1. The rearrangement of matrix molecules permits integrin clustering and the dynamic remodeling of focal Adhesions. This dynamism induces transient fluctuations in local membrane tension, the frequency and amplitude of which may fall precisely within the sensitivity window of Piezo1. Piezo1 knockdown experiments confirm that without this channel, cells lose the ability to discriminate between matrix stress relaxation rates. Current biomaterials often struggle to independently regulate elastic modulus (stiffness) and loss modulus (viscosity). Future efforts must develop next-generation hydrogel systems with orthogonal tunability (e.g., via orthogonal click chemistry or supramolecular assembly) to precisely define the response threshold of Piezo1 to viscous damping. Future research should leverage high-speed Lattice Light-Sheet Microscopy combined with novel membrane tension probes to observe the “flickering” mode of Piezo1 on viscoelastic substrates at the millisecond scale. A key question is whether a specific “frequency encoding” exists that distinguishes the Piezo1 opening patterns induced by fast-relaxing substrates from those on static stiff substrates, thereby activating distinct combinations of downstream transcription factors (Yang S. et al., 2022; Lewis and Grandl, 2015). Finally, this perspective offers new insights into diseases like Non-union or Osteoarthritis. The driver may not be merely tissue “softening” or “stiffening”, but a pathological alteration in stress relaxation properties (e.g., aged cartilage becoming too “purely elastic” and lacking dissipation capacity, or fibrotic tissue becoming excessively “viscous”). If Piezo1 sensors are miscalibrated by these changes, stress relaxation rates could serve as novel biomechanical biomarkers for disease diagnosis (Dai et al., 2025; Knecht et al., 2025).

### 6.2 The structural pharmacology “black box” and AI-empowered rational design

Despite the success of Cryo-Electron Microscopy (Cryo-EM) in resolving the three-bladed propeller architecture of Piezo1 and revealing its “nanodome” conformation within lipid bilayers, a significant challenge remains: current structural data are predominantly static snapshots obtained in non-physiological environments, such as detergents or nanodiscs. This limitation creates a massive “structural black box” for Piezo1-targeted drug development. The “Force-from-lipids” theory underscores the decisive influence of membrane composition on channel function. Osteocyte membranes are enriched with cholesterol and specific phosphoinositides (PIPs), which not only regulate membrane bending stiffness but may also bind directly to the Blade or Anchor domains. For instance, aging-associated alterations in osteocytic membrane lipids (e.g., cholesterol depletion or oxidized lipid accumulation) may drive Piezo1 into a “desensitized” or “long-term inactivated” state (Vasileva and Chubinskiy-Nadezhdin, 2023; Stommen et al., 2024), providing a structural basis for the blunt mechanosensitivity observed in the aged skeleton. The advent of AlphaFold 3 offers a dawn for overcoming the challenges of targeting the massive Piezo1 complex (>2,500 amino acids per monomer). Unlike its predecessors, AlphaFold 3 possesses the capability to predict protein-ligand complex structures and conformational ensembles (Fang et al., 2025). The current agonist, Yoda1, functions as a “molecular wedge” that inserts between the transmembrane domains to lower the mechanical activation threshold (Jiang et al., 2023). By leveraging AlphaFold 3 in conjunction with large-scale Molecular Dynamics (MD) simulations, we can now predict the binding energies of billions of small molecules against distinct Piezo1 conformational states. This facilitates the screening of functional “biased agonists”—for example, agents that enhance Ca^2+^ permeability without triggering rapid inactivation, or those that bind exclusively within specific lipid environments (e.g., the osteocyte membrane). Looking forward, Cryo-Electron Tomography (Cryo-ET) holds the promise of resolving the supramolecular structure of Piezo1 complexed with the cytoskeleton and extracellular matrix (collagen/fibronectin) *in situ* within intact osteocyte dendrites (Mulhall et al., 2023). This would definitively resolve the debate regarding the relative weights of the “Force-from-lipids” versus “Force-from-filaments/tethers” models in bone tissue. Given that pathological mechanical loading (e.g., high-frequency vibration or sustained compression) often drives the channel into a deep, difficult-to-arouse inactivated state, developing “inactivated state destabilizers”—molecules specifically designed to prevent or reverse this inactivation—represents the “Holy Grail” for treating senile osteoporosis.

### 6.3 The dilemma of targeted delivery: anchoring to bone within a systemic network

Piezo1 expression is characterized by extreme ubiquity. Beyond its role as the guardian of the skeleton, it functions as a regulator of red blood cell volume, a sensor of endothelial shear stress, a maintainer of lymphatic valve competence, and a determinant of neural stem cell fate (Lei et al., 2024). Consequently, current agonists (e.g., Yoda1) or inhibitors (e.g., GsMTx4) suffer from a critical lack of tissue specificity. While systemic administration increases bone mass in murine models, it entails unacceptable risks of off-target effects, including blood pressure fluctuations, thrombosis, and hemolysis. Therefore, precisely confining “mechanical signal interventions” to the skeletal microenvironment remains the single greatest bottleneck for clinical translation. Current bone-targeting strategies predominantly rely on the affinity of bisphosphonates or poly-aspartic acid peptides for hydroxyapatite. However, this “mineral-binding” strategy has inherent flaws: drugs become sequestered on the mineralized surface and may fail to effectively access Piezo1 channels located on the membranes of osteocytes deeply buried within the lacunar-canalicular network (LCN) (Guan et al., 2024). To overcome these barriers, three innovative directions are proposed:1. Exosome Engineering (Bio-inspired Delivery): Developing exosomes derived from BMSCs or osteoblasts as natural nanocarriers for Piezo1 modulators (mRNA, siRNA, or small molecules). By genetically engineering the exosome surface to display aptamers or antibody fragments targeting osteocyte-specific membrane proteins (e.g., Sclerostin or Connexin 43 hemichannels), true “cellular-level” targeting can be achieved. 2. Mechano-Responsive Delivery (Smart Carriers): Exploiting the unique mechanical milieu of bone tissue—specifically, high fluid shear stress (FSS) and hydrostatic pressure—as an endogenous trigger. Shear-sensitive liposomes or polymeric nanocapsules can be designed to deform and release their payload *only* within the fluid-active environment of the bone canaliculi, thereby remaining stable in static, non-osseous tissues. 3. Magneto-Mechanical Actuation (Wireless Control): Functionalizing Magnetic Nanoparticles (MNPs) to specifically bind to the extracellular domain of Piezo1. Upon application of a low-frequency external magnetic field, these particles generate micro-torque or tensile force to physically “pull open” the channel. This strategy completely circumvents the systemic distribution issues of chemical agonists, realizing a non-invasive, spatiotemporally controllable, and “wireless” paradigm for bone regeneration.

### 6.4 Multi-physics coupling: a panoramic view beyond single mechanical forces

Bone operates as a sophisticated multi-physics coupled organ. It is subjected not only to mechanical forces but also integrates piezoelectricity, streaming potentials, and thermal effects. Traditional Piezo1 research has often isolated mechanical stimuli (compression, stretch) from electrical, thermal, or magnetic fields. However, a frontier perspective emerging in 2025 posits that *in vivo* Piezo1 activation is a process of “Multi-physics Synergism”. Research indicates that while Piezo1 exhibits only weak voltage-gating properties, its mechanosensitivity can be significantly modulated by endogenous electric fields generated within the bone matrix. Consequently, motion-induced piezoelectric potentials may act as a “priming background”, effectively lowering the activation threshold of Piezo1 to fluid shear stress. Understanding this coupling mechanism provides a solid molecular rationale for the combined application of Pulsed Electromagnetic Field (PEMF) therapy and mechanotherapy. LIPUS serves as a standard clinical therapy for fracture healing, yet its precise mechanism has long remained elusive. Recent patch-clamp recordings and finite element simulations have now confirmed that LIPUS directly activates Piezo1 via acoustic streaming-induced shear stress or microbubble cavitation effects. The future challenge lies in establishing a quantitative atlas of “Acoustic Parameters–Piezo1 Kinetics”. The goal is to develop specific ultrasound frequencies capable of resonating with the Piezo1 channel. This would enable non-invasive “Sono-genetics” (acoustically targeted regulation) of deep bone tissues, representing a potentially revolutionary modality for treating refractory conditions such as non-union or deep osteonecrosis.

### 6.5 Spatial mechanomics: building the “google maps” of skeletal mechanics

To truly decipher the role of Piezo1 in skeletal development and regeneration, we must transcend the spatiotemporal resolution limits of traditional “bulk sequencing”. Fracture healing is an inherently highly heterogeneous process; cells in the callus center (characterized by hypoxia and high strain) reside in a mechanical microenvironment vastly distinct from those at the periphery (normoxia and low strain). The emergence of Spatial Mechanomics offers the ultimate tool to resolve this complexity. By integrating micron-resolution spatial transcriptomics technologies (e.g., Visium HD, Xenium) with *in vivo* micro-CT and Finite Element Analysis (micro-FE), researchers can now precisely register the gene expression profile of every single cell with its local mechanical parameters, such as local strain and fluid flow velocity. Future efforts will focus on constructing a 4D fracture healing atlas, tracking the dynamic trajectory of Piezo1 and its downstream pathways as they evolve in concert with the mechanical environment. This endeavor aims to reveal the existence of specific “mechanical therapeutic windows”—critical temporal phases where targeted intervention yields the maximum therapeutic efficacy.

### 6.6 Conclusion: ushering in the new era of “mechano-mimetics”

In summary, the discovery of Piezo1 and the elucidation of its core functions in bone tissue constitute a cornerstone of modern bone biology. It not only provides a perfect molecular interpretation of the century-old Wolff’s Law but also unveils an exquisitely sophisticated cellular signal integration network—the “Piezo1 Mechanical Signal Rheostat”. Through multidimensional axes—including the “ionic cascade”, “cytoskeletal-nuclear mechanics”, “metabolic reprogramming”, and the “inflammatory-stress response”—this rheostat converts millisecond-scale physical stimuli into enduring biochemical instructions that dictate cell fate. However, Piezo1 is not a simple binary switch; it functions as a highly context-dependent regulatory hub. Its functionality is collectively constrained by the membrane lipid environment, cytoskeletal tension, viscoelastic matrix properties, and the multi-physics milieu. This complexity presents both a challenge and an opportunity. Looking forward, with atomic-level resolution in structural biology, leaps in AI-aided drug design, the maturation of spatial omics, and innovations in smart biomaterials, we stand on the threshold of the “Mechano-mimetics” era. Future orthopedic treatments will no longer be limited to passive measures like calcium supplementation or fixation, but will actively modulate the cellular mechanosensing system.

1. “Molecular Exercise Pills”: Utilizing specific Piezo1 agonists to mimic exercise-induced osteogenic signals, offering a solution for disuse bone loss in bedridden patients or astronauts.
2. Smart Regenerative Implants: Deploying 4D-printed scaffolds capable of responding to *in vivo* mechanical changes to release Piezo1 modulators on demand, thereby achieving a dynamic “mechanical dialogue” with host cells.
3. Precision Physical Therapy: Developing personalized acoustic, electrical, and magnetic combination therapies based on Piezo1 resonance frequencies to achieve non-invasive, precise intervention at lesion sites.

The exploration of Piezo1 has not only decoded the “mechanical language of life” but also armed humanity with unprecedentedly powerful weapons to conquer obstinate diseases such as osteoporosis, osteoarthritis, and impaired fracture healing. From mechanosensation to cell fate, and ultimately to clinical cure, this cross-scale scientific journey may provide promising avenues for mechanism-based therapeutic development.
